# Incidence of health-care-associated infections in long-term care facilities in nine European countries: a 12-month, prospective, longitudinal cohort study

**DOI:** 10.1016/S1473-3099(25)00217-8

**Published:** 2025-11

**Authors:** Enrico Ricchizzi, Elena Sasdelli, Anna Caterina Leucci, Elisa Fabbri, Luana Caselli, Katrien Latour, Laura Int Panis, Eddy De Baets, Anne-Marie Van den Abeele, Angelo D'Ambrosio, Pete Kinross, Jaana-Marija Lehtinen, Côme Daniau, Adeline Paumier, Costanza Vicentini, Kassiani Mellou, Auguste Salnaite, Murielle Weydert, Kati Halonen, Anna Rozanska, Pilar Gallego-Berciano, Carl Suetens, Tommi Kärki

**Affiliations:** a Settore Innovazione nei Servizi Sanitari e Sociali, Regione Emilia-Romagna, Bologna, Italy; b Sciensano, Ixelles, Belgium; c St-Lucas Hospital, Bruges, Belgium; d European Centre for Disease Prevention and Control, Solna, Sweden; e Finnish Institute for Health and Welfare, Helsinki, Finland; f Santé Publique France, Paris, France; g Department of Public Health and Pediatrics, University of Turin, Turin, Italy; h National Public Health Organization, Athens, Greece; i Institute of Hygiene, Vilnius, Lithuania; j Directorate of Health, Luxembourg City, Luxembourg; k National Institute for Public Health and Environment, Centre for Infectious Disease Epidemiology and Surveillance, Bilthoven, Netherlands; l Department of Microbiology, Faculty of Medicine, Jagiellonian University Medical College, Krakow, Poland; m National Center of Epidemiology, Institute of Health Carlos III, Madrid, Spain

## Abstract

**Background:**

The number of older people in need of long-term care is increasing, and health-care-associated infections (HAIs) are a major cause of morbidity and mortality for residents of long-term care facilities (LTCFs). This study, organised by the European Centre for Disease Prevention and Control (ECDC), provided data on the incidence of HAIs and related adverse outcomes in LTCFs in European countries, supplementing the available estimates from repeated point prevalence surveys conducted by the ECDC.

**Methods:**

In this longitudinal, prospective cohort study, we analysed all HAIs collected in a convenience sample of residents from 65 LTCFs (including general nursing homes, residential homes, and mixed facilities) in nine EU or European Economic Area (EEA) countries (Belgium, Finland, France, Italy, Lithuania, Luxembourg, the Netherlands, Poland, and Spain) over 12 months. Eligible residents were those expected to stay in the LTCF for at least the entire study period. Data were collected with three questionnaires: an institutional questionnaire, a residents' questionnaire, and an HAI questionnaire. HAIs were defined according to standard ECDC criteria. The primary outcome was HAI incidence. Incidence measures, estimated using generalised estimating equation models to account for sample heterogeneity, were percentages of each type of HAI, numbers of HAIs per 100 LTCF residents (ratio), and numbers of HAIs per 1000 resident-days (incidence rate).

**Findings:**

HAIs were analysed in 3029 residents of LTCFs between Jan 1–May 4, 2022, and Jan 1–May 12, 2023. The mean age of study participants was 80·9 years (SD 14·6), including 960 (31·7%) men and 2069 (68·3%) women. 3763 HAIs were recorded, with at least one HAI identified in 1717 (57%) of 3029 residents. There were 124·2 HAIs (95% CI 118·6–129·9) per 100 residents and 1·8 HAIs (0·9–3·3) per 1000 resident-days. 160 (4·3% [95% CI 3·9–5·4]) HAIs led to hospitalisation, and 154 (4·5% [2·5–4·8]) were associated with death. Respiratory tract infections (RTIs) were the most frequent type of infection (n=1080, 28·9% [95% CI 27·3–30·5]), including pneumonia (n=279, 7·3% [6·4–8·3]) and other lower RTIs (n=394, 10·7% [9·6–11·8]), followed by urinary tract infections (UTIs; n=743, 18·7% [17·2–20·3]). RTIs showed the highest incidence of mortality (n=85, 2·3% [95% CI 1·8–2·8] of all HAIs). Severe cases of COVID-19 (n=72, 1·9% [95% CI 1·5–2·4] of all HAIs) were less frequent than mild or moderate cases (n=615, 16·0% [14·9–17·1] of all HAIs).

**Interpretation:**

This study shows the high incidence of HAIs among LTCF residents in EU or EEA countries, with more than one in two residents experiencing at least one HAI, and with RTIs and UTIs accounting for almost half of all observed HAIs.

**Funding:**

European Centre for Disease Prevention and Control.

## Introduction

Reflecting gains in life expectancy, the ageing population of Europe is driving an increased demand for long-term care. Although there are differences between countries in national health-care systems, culture, and demographics, long-term care in Europe generally consists of a range of medical care, personal care, and assistance services that are provided to reduce or manage a deterioration in health status for individuals who are no longer able to conduct activities of daily living without assistance.^1^ In EU and European Economic Area (EEA) countries, most long-term care facilities (LTCFs) are for older people and are general nursing homes, residential homes, or mixed-type facilities. Residents are mostly medically stable and do not require specialised care or invasive procedures but are supervised 24 h a day with high-skilled nursing care.^2^

The number of people aged 65 years and older in Europe exceeded 90 million in 2019 and is projected to increase substantially in the coming decades, reaching almost 130 million by 2050.^3^ Over this period, the number of people aged 65–74 years is expected to increase by 17%, and the number aged 75–84 years is expected to increase by 56%.^3^ As a result, the number of people potentially in need of long-term care is also estimated to increase from 30 million in 2019 to almost 40 million by 2050.^4^ These growing care needs are reflected in the number of LTCF beds relative to population size, which increased, on average, between 2012 and 2025^5^ and in a preprint was projected to increase further in the coming decades.^6^ The total number of LTCF beds recorded in the EU in 2022 was approximately 3·5 million,^4^, ^5^ indicating that LTCFs are becoming a substantial component of health-care delivery in European countries.

**Research in context**

**Evidence before this study**

Since 2008, the European Centre for Disease Prevention and Control (ECDC) has organised repeated point prevalence surveys of health-care-associated infections (HAIs) and antimicrobial use in European long-term care facilities (LTCFs)—ie, HALT surveys. Although HALT surveys have provided valuable insights, from point prevalence survey data alone, it is not possible to estimate the annual number of HAI episodes or the number or burden of HAI-related adverse events, such as hospitalisations and deaths. We searched PubMed with the search terms (“healthcare-associated infections” OR “nosocomial infections” OR “infections”) AND (“long-term care facilities OR “long-term care” OR “nursing homes”) AND (“surveillance” OR “incidence” OR “burden”) for articles reporting the incidence of HAIs in the LTCF setting published between Jan 1, 2005, and Dec 31, 2024. We found prospective surveillance studies conducted in single countries, mostly in a small number of facilities of one type (eg, nursing homes), over a short time period (eg, months), and, in some cases, focusing on specific HAIs or subsets of HAIs. No study systematically examined the incidence of HAIs and related adverse outcomes at the level of individual LTCF residents, and, given the differences in methods used, the findings of these studies are difficult to compare.

**Added value of this study**

To our knowledge, this study is the first longitudinal surveillance survey to estimate the incidence of HAIs and HAI-related hospitalisations and mortality over a 12-month period (2022–23) in a large sample of LTCFs of different sizes and types (ie, general nursing homes, residential homes, and mixed-type facilities) in Europe. Conducted as a part of ECDC surveillance activities in LTCFs, this study provides a comprehensive account of the effect of a wide range of HAIs, including COVID-19, on more than 3000 residents of 65 LTCFs in nine European countries. Despite some variability in the implementation of the survey due to heterogeneity in the characteristics of the participating LTCFs, the use of an integrated standardised methodology for data collection ensured reasonable generalisability of results across European countries. More than half of the LTCF residents in the study contracted at least one HAI over the surveillance period, with an estimated overall incidence rate of 1·8 HAIs (95% CI 0·9–3·3) per 1000 resident-days. Respiratory tract infections and urinary tract infections showed the highest incidence rates (1·1 [1·0–1·3] and 0·8 [0·6–1·0] per 1000 resident-days, respectively); these HAIs were also associated with the highest hospitalisation and mortality rates.

**Implications of all the available evidence**

This study complements prevalence estimates available from the HALT surveys with longitudinal data on the incidence of HAIs and HAI-related adverse outcomes, providing essential information for estimating the overall burden of HAIs in LTCFs in Europe. It also supports findings from previous national and subnational reports, showing a high incidence of HAIs among LTCF residents, and provides results on the risk of HAIs in LTCFs in Europe. Further investigations are needed to analyse risk factors for HAIs, with the aim of facilitating targeted local interventions to prevent and control HAIs and HAI-related negative health outcomes.

Health-care-associated infections (HAIs) are a major cause of morbidity and mortality in older individuals living in LTCFs, who might be frail and have multiple underlying health conditions.^7^, ^8^ Since 2008, the European Centre for Disease Prevention and Control (ECDC) has done repeated point prevalence surveys of HAIs and antimicrobial use in European LTCFs (ie, HALT surveys).^2^, ^9^, ^10^ Based on the results of the point prevalence surveys organised in 2016–17 (HALT-3),^2^ the country-weighted prevalence of residents with at least one HAI was 3·9%, pointing to LTCF residents having a risk for HAIs similar to that of patients in acute care hospital settings.^11^ In 2016–17, the most frequently reported HAIs were respiratory tract infections (RTIs), urinary tract infections (UTIs), and skin and soft-tissue infections. Moreover, LTCF residents have been at higher risk of adverse outcomes related to COVID-19 than have older people living in the community.^12^, ^13^, ^14^, ^15^ Most national COVID-19 vaccination programmes have prioritised residents in LTCFs to mitigate the spread of SARS-CoV-2 and its impact in LTCFs. However, with the emergence of SARS-CoV-2 variants of concern capable of evading vaccine-induced immunity, breakthrough infections can occur.^16^, ^17^

As a part of ECDC surveillance activities in LTCFs,^2^, ^9^, ^10^, ^16^, ^18^ this study aimed to complement the available point prevalence survey estimates of HAIs with longitudinal data on the incidence of HAIs, including COVID-19, in LTCFs in Europe and to describe HAI-related hospitalisations and mortality. Such data are essential to estimate the burden of HAIs in European LTCFs.

## Methods

### Study design

This prospective, longitudinal cohort study recruited LTCFs to participate in a survey aiming to collect data on HAIs for 12 months, starting during the first half of 2022 and ending in the first half of 2023. LTCFs in all EU and EEA countries were invited through the Healthcare-Associated Infections Surveillance Network, coordinated by the ECDC with the support of an external contractor (ie, a consortium of Sciensano [Brussels, Belgium] and Agenzia Sanitaria e Sociale Regionale—Emilia Romagna [Bologna, Italy]; framework contract ECDC/2020/006). Eligible LTCFs were general nursing homes, residential homes, and mixed-type facilities (criteria are in the study protocol, appendix p 18), preferably with pre-existing continuous HAI surveillance and a median length of stay for residents of about 12 months or longer. Other types of facilities or specific wards or departments within larger LTCFs were also eligible (excluding long-term hospital care wards, hotels without any kind of nursing care, sheltered care houses, day centres, home-based centres, and protected living). Eligible residents were expected to stay in the LTCF for at least the entire study period. New residents who entered the LTCF while the study was already underway were excluded. Eligible residents who were discharged or died during the study period were censored at discharge or death.

Whether ethical approval was required for the survey depended on the country, with some countries requiring approval from an ethics committee (appendix p 1) as well as written informed consent from the residents (or their proxies). Data confidentiality was ensured by the assignment of a unique survey identification code to each LTCF and each individual resident. Residents were not subject to any interventions as part of the study.

### Survey administration procedures

In each country, one or more national survey coordinators were responsible for the recruitment of LTCFs and organisation of the study (appendix p 29). The data collectors were local health-care professionals or external data collectors (ie, professionals experienced in HAI surveillance or national survey coordinators), with both local and external data collectors trained on the survey protocol during online meetings and case study workshops. The data collectors received three survey questionnaires in a printable format (appendix pp 32–37). The first was an institutional questionnaire (one per LTCF, administered at the beginning of the study), which collected information on LTCF characteristics (eg, type, number of beds, presence or absence of any coordinating medical physician, and presence or absence of HAI surveillance programme or routine diagnostic laboratory tests). The second was a residents' questionnaire (one per resident), which collected information on general demographics (eg, year of birth and sex), date of admission to the LTCF, risk factors for HAIs (eg, disorientation, mobility, incontinence, and presence of urinary or vascular catheter [or both]), comorbidities (assessed by the Charlson Comorbidity Index),^19^ SARS-CoV-2 infection and COVID-19 vaccination history at the start of the study, whether there were any temporary discharges from the LTCF during the study, the end date of follow-up, and status (ie, alive or deceased) at the end of follow-up. These data were collected directly from the residents and from their medical records. The population was described by its demographic and clinical characteristics, with sociocultural factors (eg, ethnicity or educational qualifications) or economic factors (eg, income) not considered in this study. The third questionnaire collected information about HAIs developed by any resident during the follow-up period (with one provided for each identified HAI), including infection site, duration, outcome (eg, alive or deceased with HAI as sole cause, contributory cause, part of the causal sequence, or with no contribution), and if the infection was diagnosed in hospital; provision of the antimicrobial resistance profile was optional.

Data from the paper questionnaires were entered into standardised Microsoft Excel spreadsheets directly by the LTCF staff or by the national survey coordinators (who provided a monthly check for missing data and errors through direct contact with LTCF staff). National spreadsheets were then aggregated into a single European database by the study coordinator and checked again by ER for missing data and errors. Countries were required to report zero HAIs if no infections were identified during the 1-month period.

### Identification of HAIs

Residents presenting with signs or symptoms of an HAI or with a positive laboratory test for COVID-19 were identified and registered through the HAI questionnaire. Infections were considered as HAIs and included in the survey if they were acquired within an LTCF (current or other LTCF) or during a temporary discharge, with symptoms of infection occurring at least 2 days after readmission to the LTCF.

To assign a unique identification code to each HAI (appendix p 25), data collectors used clinical decision-making algorithms based on McGeer's criteria for the surveillance of HAIs in LTCFs^20^ (see also appendix pp 38–44), with ECDC case definitions adapted from those of the US Centers for Disease Control and Prevention and the Society for Healthcare Epidemiology of America.^18^, ^21^ The COVID-19 case definition was based on the positive result of a laboratory test. A code list of microorganisms (appendix pp 45–48) was also provided in the study protocol for registration of the detected microorganisms and (optionally) their antimicrobial resistance profiles.

### Outcomes

The primary outcome was incidence of HAIs in LTCFs over the 12-month follow-up period, addressed by considering the following endpoints: percentage of residents with at least one HAI; percentage of HAIs by type out of all HAIs; ratio—ie, average number of HAIs per 100 LTCF residents, computed as the number of HAIs divided by the total number of residents multiplied by 100; and incidence rate of HAIs—ie, number of HAIs per 1000 resident-days.

Secondary outcomes were HAI-related hospitalisations and HAI-related deaths, estimated with the same incidence metrics as used for the primary outcome. HAI-related hospitalisations were defined as those occurring between the onset and end of the HAI. The end of the HAI was established by the following (in order of priority): end of the treatment; declaration of recovery by the attending physician; complete remission of clinical signs and symptoms; date of first negative sample; or date estimation, if none of the previous information was available. HAI-related deaths were defined as those with an HAI registered either as the sole cause of death, part of the causal sequence of events leading to death, or as a contributory cause of death.^22^ The numbers of hospitalisations and deaths that occurred within 7 days and 30 days of the onset of HAI were also assessed.

### Statistical analysis

Overall, we assumed that the LTCFs participating in the study had the same characteristics as the LTCFs surveyed in HALT-3.^18^ Our planned sample consisted of five to eight countries, with a total number of residents of approximately 1250–2000. For feasibility, no formal sample size calculation was used for this study.

To estimate percentages of HAIs, HAI-related hospitalisations, and HAI-related deaths caused by each type of HAI, we used binomial generalised estimating equation (GEE) models with an intercept term only. The dependent variables were binary, indicating the presence or absence of a specific type of HAI or whether each type of HAI resulted in hospitalisation or death. The reported values were calculated as odds/(1 + odds) and correspond to the estimated probabilities from the model, which when multiplied by 100, can be interpreted as percentages.

To estimate the incidence ratios of HAIs, HAI-related hospitalisations, and HAI-related deaths, we used Poisson's GEE models with an intercept term only. The dependent variables were counts, indicating the number of each type of HAI or the number of each type of HAI that resulted in hospitalisation or death. The reported values are exponentials of the estimate.

To estimate the incidence rates of HAIs, HAI-related hospitalisations, and HAI-related deaths, we used negative binomial GEE models with an intercept term only, introducing the log of follow-up days as an offset term. The dependent variables were counts, indicating the number of each type of HAI or the number of each type of HAI that resulted in hospitalisation or death.

For all models, repeated measures on residents and clustering of residents within the same LTCF were considered by using a compound symmetry covariance structure in the GEE models. This approach assured robustness for within-cluster correlation and accounted for heterogeneity across countries and LTCFs. This strategy allowed us to mitigate the effect of differences in population characteristics by estimating marginal effects across clusters. Data were analysed using SAS 8.3 update 6 (8.3.6.200).

### Role of the funding source

The ECDC coordinated the work on the initial study design and provided comments on the data analysis and the final report. The ECDC did not have a direct role in data collection and did not pose any restrictions on data interpretation by the authors.

## Results

Of the initial sample (69 LTCFs and 3279 residents in ten EU or EEA countries invited), 65 LTCFs and 3029 residents (59% more than the planned sample) in nine countries (Belgium, Finland, France, Italy, Lithuania, Luxembourg, the Netherlands, Poland, and Spain) completed the follow-up survey at 12 months. Start dates ranged from Jan 1, 2022, to May 4, 2022, and end dates ranged from Jan 1, 2023, to May 12, 2023 (exact start and end dates by country are in the appendix [p 1]). Only one country of the initial ten did not complete the 12 months of follow-up, and their partial data were not included in the analyses.

The clinical and demographic characteristics of the final study population (LTCFs and residents per country) are in table 1. Of the 3029 LTCF residents, 2069 (68%) were women and 960 (32%) were men; the mean age of the study participants was 80·9 years (SD 14·6). 1385 (46%) residents were wheelchair-bound or bedridden, and 2025 (67%) had incontinence. The mean Charlson Comorbidity Index score was 2·9 (SD 2·7), indicating that residents were affected by moderate-to-severe comorbidities. The distribution of comorbidities per participating country is provided in the appendix (p 7). 28 (43%) of 65 LTCFs reported that they had an HAI surveillance system in place.

**Table 1:** Clinical and demographic characteristics of the study population

		**Belgium**	**Finland**	**France**	**Italy**	**Lithuania**	**Luxembourg**	**Netherlands**	**Poland**	**Spain**	**Total**
LTCFs		4	15	10	24	5	2	3	1	1	65
LTCF residents		260	344	702	395	368	153	275	269	263	3029
Age, years		81·2 (11·0)	84·9 (7·4)	87·1 (8·7)	85·0 (10·0)	61·3 (14·8)	59·3 (22·6)	83·3 (10·0)	84·1 (10·2)	87·5 (8·5)	80·9 (14·6)
Sex											
	Female	174 (67%)	235 (68%)	516 (74%)	278 (70%)	188 (51%)	73 (48%)	179 (65%)	210 (78%)	216 (82%)	2069 (68%)
	Male	86 (33%)	109 (32%)	186 (26%)	117 (30%)	180 (49%)	80 (52%)	96 (35%)	59 (22%)	47 (18%)	960 (32%)
Disorientation											
	Mild	52 (20%)	71 (21%)	102 (15%)	105 (27%)	41 (11%)	28 (18%)	53 (19%)	49 (18%)	41 (16%)	542 (18%)
	Moderate	49 (19%)	111 (32%)	143 (20%)	65 (16%)	67 (18%)	19 (12%)	55 (20%)	80 (30%)	51 (19%)	640 (21%)
	Severe	45 (17%)	135 (39%)	345 (49%)	77 (19%)	57 (15%)	13 (8%)	50 (18%)	101 (38%)	156 (59%)	979 (32%)
	Missing data	0	6 (2%)	2 (<1%)	1 (<1%)	0	1 (<1%)	6 (2%)	4 (1%)	0	20 (<1%)
Mobility											
	Ambulant	178 (68%)	166 (48%)	397 (57%)	131 (33%)	312 (85%)	118 (77%)	139 (51%)	27 (10%)	163 (62%)	1631 (54%)
	Wheelchair	75 (29%)	93 (27%)	201 (29%)	250 (63%)	34 (9%)	35 (23%)	124 (45%)	117 (43%)	85 (32%)	1014 (33%)
	Bedridden	7 (2·7%)	85 (25%)	102 (15%)	14 (4%)	22 (6%)	0	9 (3%)	117 (43%)	15 (6%)	371 (12%)
	Missing data	0	0	2 (<1%)	0	0	0	3 (1%)	8 (3%)	0	13 (<1%)
Incontinence		168 (64%)	283 (82%)	517 (74%)	279 (71%)	127 (35%)	53 (35%)	144 (52%)	247 (92%)	207 (79%)	2025 (67%)
	Missing	0	0	5 (<1%)	0	0	0	22 (8%)	10 (4%)	0	37 (1%)
Urinary catheter		10 (4%)	22 (6·4)	11 (2%)	24 (6%)	3 (<1%)	9 (6%)	35 (13%)	39 (14%)	4 (2%)	153 (5%)
	Missing data	0	0	6 (<1%))	0	0	0	6 (2%)	27 (10%)	0	39 (1%)
Vascular catheter		1 (<1%)	1 (<1%)	2 (<1%)	7 (2%)	0	0	1 (<1%)	18 (7%)	2 (<1%)	32 (1%)
	Missing data	0	0	6 (<1%)	0	0	0	7 (3%)	32 (12%)	0	45 (1%)
Charlson Comorbidity Index		2·8 (2·4)	2·5 (2·3)	3·5 (2·8)	2·9 (2·2)	3·1 (3·1)	1·3 (2·0)	1·2 (1·4)	3·4 (2·5)	2·8 (3·0)	2·9 (2·7)

Data are n, n (%), or mean (SD). Percentages refer to the sample of each country. LTCF=long-term care facility.

**Figure 1:**
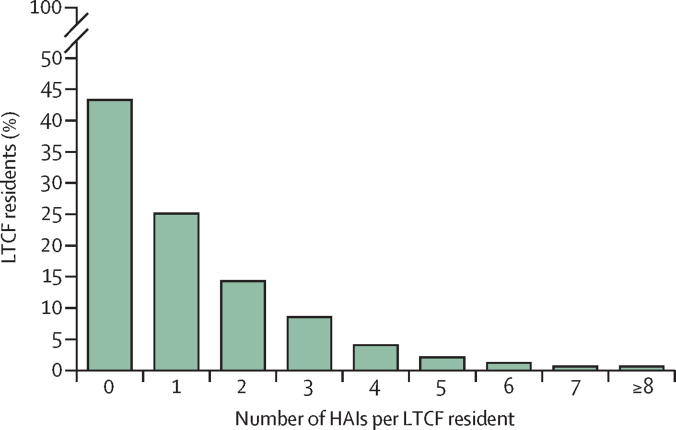
Percentage distribution of the number of HAIs per LTCF resident HAI=health-care-associated infection. LTCF=long-term care facility.

**Figure 2:**
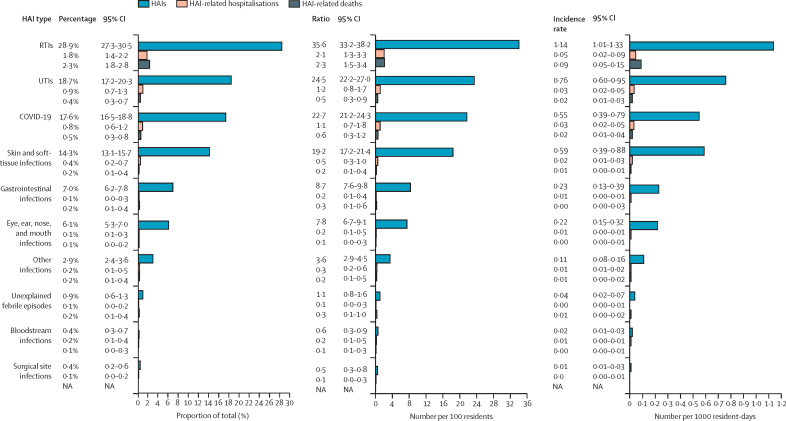
Estimated percentages, ratios per 100 residents, and rates per 1000 resident-days of HAIs, HAI-related hospitalisations, and HAI-related deaths, by type of HAI Results from intercept-only generalised estimating equation models with an exchangeable correlation structure. Percentages and incidence rates were estimated with negative binomial models (N=3763 HAIs). Ratios were estimated with Poisson models (N=3029 residents). All models were adjusted for repeated measures on residents and clustering in long-term care facilities. There were no deaths related to surgical site infections . The x-axis in the first graph only runs from 0 to 30% to better display the differences between the groups at smaller percentages. HAI=health-care-associated infection. NA=not applicable. RTI=respiratory tract infection. UTI=urinary tract infection.

The number of HAIs recorded during the 12-month follow-up period was 3763, with 1717 (57%) of 3029 residents having contracted at least one HAI and 969 (32%) having contracted more than one HAI. Figure 1 shows the percentage distribution of the number of HAIs per resident. The estimated ratio of HAIs was 124·1 (95% CI 118·6–129·9) per 100 residents, and the incidence rate was 1·8 (95% CI 0·9–3·3) per 1000 resident-days. The most frequent HAIs were RTIs (n=1080, estimated to account for 28·9% [95% CI 27·3–30·5] of all HAIs), followed by UTIs (n=743, 18·7% [17·2–20·3] of all HAIs; figure 2; appendix p 8). Among the RTIs, there were 279 (7·3% [6·4–8·3] of total HAIs) registered episodes of pneumonia and 394 (10·7% [9·6–11·8] of total HAIs) of other lower RTIs. COVID-19 (n=687, 17·6% [16·5–18·8] of total HAIs) mostly manifested as mild or moderate disease (n=615, 16·0% [14·9–17·1] of total HAIs), with only 72 severe cases (1·9% [1·5–2·4] of all HAIs; appendix p 8). Crude numbers, percentages, ratios, and incidence rates of HAIs, by type of HAI and country are in the appendix (p 9).

160 HAIs led to hospitalisation, accounting for 4·3% (95% CI 3·9–5·4) of all HAIs. The ratio of HAI-related hospitalisations was 5·0 (95% CI 4·1–7·6), per 100 residents, and the incidence rate was 0·09 (95% CI 0·05–0·21) per 1000 resident-days. The highest incidence of hospitalisations was observed for RTIs (n=63, 1·8% [95% CI 1·4–2·2] of all hospitalisations), followed by UTIs (n=32, 0·9% [0·7–1·3]). 27 (0·8% [0·6–1·2]) HAI-related hospitalisations were due to COVID-19 (figure 2; appendix p 10). Numbers, percentages, ratios, and incidence rates of hospitalisation, by type of HAI and country are in the appendix (p 11).

Table 2 shows the ratios of hospitalisations per 100 residents at 7 days and 30 days after the onset of an HAI. The number of hospitalisations was higher at 30 days (n=609) than at 7 days (n=503), with slight increases from 7 days to 30 days for most types of HAI. Hospitalisations occurred most frequently after the onset of RTIs, UTIs, skin and soft-tissue infections, and COVID-19.

**Table 2:** Ratios of hospitalisations per 100 residents at 7 days and 30 days after HAI onset, by HAI type

	**7-day hospitalisations**			**30-day hospitalisations**		
	n	Crude ratio	Estimated ratio (95% CI)	n	Crude ratio	Estimated ratio (95% CI)
RTIs	158	5·2	4·4 (3·9–5·5)	202	6·7	5·1 (4·3–5·7)
UTIs	110	3·6	2·6 (2·1–3·7)	133	4·4	2·9 (2·3–3·6)
Skin and soft-tissue infections	72	2·1	1·6 (1·2–2·2)	86	2·8	1·9 (1·4–2·4)
COVID-19	57	1·9	1·6 (1·2–2·0)	72	2·4	2·0 (1·6–2·5)
Gastrointestinal infections	35	1·2	0·9 (0·6–1·2)	38	1·3	1·0 (0·7–1·3)
Eye, ear, nose, and mouth infections	26	0·9	0·7 (0·4–1·0)	32	1·1	0·8 (0·6–1·2)
Other infections*	19	0·6	0·5 (0·3–0·8)	21	0·7	0·6 (0·4–0·9)
Surgical site infections	10	0·3	0·3 (0·1–0·5)	10	0·3	0·3 (0·1–0·5)
Bloodstream infections	10	0·3	0·3 (0·2–0·5)	11	0·4	0·3 (0·1–0·5)
Unexplained febrile episodes	4	0·1	0·1 (0·0–0·3)	4	0·1	0·1 (0·0–0·3)
All	501	16·5	14·1 (10·9–18·1)	609	20·1	18·8 (13·6–22·3)

HAI=health-care-associated infection. RTI=respiratory tract infection. UTI=urinary tract infection.

* Includes HAIs that did not meet any specific case definitions used in the study.

Of the 664 deaths recorded during the 12-month follow-up period, 142 (21·4%) were attributed to one or more concomitant HAIs. 154 HAIs (4·5% [95% CI 2·5–4·8] of all HAIs) were listed as causes of death, of which the most frequent were RTIs (n=85, 2·3% [1·8–2·8]), COVID-19 (n=18, 0·5% [0·3–0·8]), and UTIs (n=16, 0·4% [0·3–0·7]; appendix p 12). This incidence ranking was confirmed by the ratio of HAI-related deaths per 100 residents and rate per 1000 resident-days (figure 2; appendix p 12). The incidence rate of HAI-related deaths was 0·14 (95% CI 0·08–0·23) per 1000 resident-days. Numbers, percentages, ratios, and incident rates of deaths related to HAIs by type of HAI and country are in the appendix (p 13).

Table 3 shows the ratios of deaths per 100 residents at 7 days and 30 days after the onset of an HAI. The number of deaths within 7 days was 85 and within 30 days was 190. Deaths that occurred after the onset of an RTI accounted for most deaths within 7 days of an HAI (1·7 per 100 residents [95% CI 1·3–2·2]).

**Table 3:** Ratio of deaths per 100 residents at 7 days and 30 days after HAI onset, by HAI type

	**7-day deaths**			**30-day deaths**		
	n	Crude ratio*	Estimated ratio (95% CI)	n	Crude ratio*	Estimated ratio (95% CI)
RTIs	51	1·7	1·7 (1·3–2·2)	96	3·2	3·2 (2·6–3·9)
UTIs	9	0·3	0·3 (0·2–0·6)	30	1·0	1·0 (0·7–1·4)
COVID-19	5	0·2	0·2 (0·1–0·4)	16	0·5	0·5 (0·3–0·9)
Gastrointestinal infections	5	0·2	0·2 (0·1–0·4)	7	0·2	0·2 (0·1–0·5)
Skin and soft-tissue infections	4	0·1	0·1 (0·1–0·4)	18	0·6	0·6 (0·4–0·9)
Eye, ear, nose, and mouth infections	4	0·1	0·1 (0·1–0·4)	8	0·3	0·3 (0·1–0·5)
Unexplained febrile episodes	4	0·1	0·1 (0·1–0·4)	6	0·2	0·2 (0·1–0·4)
Other infections†	2	0·1	0·1 (0·0–0·3)	7	0·2	0·2 (0·1–0·5)
Bloodstream infections	1	0·0	0·0 (0·0–0·2)	2	0·1	0·1 (0·0–0·3)
All	85	2·8	2·0 (1·8–3·1)	190	6·3	7·0 (4·6–8·3)

RTI=respiratory tract infection. UTI=urinary tract infection.

* 11 values missing.

† Includes HAIs that did not meet any specific case definitions used in the study.

## Discussion

To the best of our knowledge, this study is the first multicentre longitudinal study on HAIs in LTCFs in Europe. Our study design, including a 12-month follow-up period and participation of nine different European countries, gives an overview of the incidence of different types of HAIs and their outcomes in residents of LTCFs in Europe. The results highlight the importance of infection prevention and control programmes in LTCFs, incorporating the planning of preventive actions, HAI surveillance, and education of staff.

Our findings support previous reports of a high incidence of HAIs among LTCF residents,^23^, ^24^, ^25^, ^26^, ^27^, ^28^ with more than one in two residents experiencing at least one HAI in our study, and with RTIs and UTIs accounting for almost half of all observed HAIs. With the exception of COVID-19, the ranking of the most common HAIs reflected the HAI distribution reported in the ECDC's point prevalence surveys in LTCFs.^2^, ^9^, ^10^ There were differences in the relative proportions of types of HAI between our study and the point prevalence surveys, which might be explained by the fact that the time windows in the point prevalence surveys (limited to April–June and September–November) did not cover entire years.

Lower RTIs other than pneumonia were the most frequently reported RTIs in the study participants and had a higher incidence than pneumonia. One of the reasons for this finding could be that, unlike the pneumonia case definition, which requires a confirmatory chest imaging test that is not always available in LTCFs, the case definition of other lower RTIs is based on clinical signs and symptoms. Therefore, compared with pneumonia, other lower RTIs were easier to detect and include in the study. In addition, common colds were likely to be recorded less frequently than were pneumonia and other lower RTIs, possibly because the clinical presentation of the common cold is often mild, with symptoms that also occur frequently in the general population and staff. Furthermore, this surveillance was done during the COVID-19 pandemic, when several containment measures were in place and countries were using different testing strategies, which could have affected the frequency of detection of upper RTIs. The interpretation of these results should also consider that surveillance was done in different respiratory virus seasons, with some LTCFs collecting data during the 2021–22 winter season and others during the 2022–23 winter season. However, the overall findings for RTIs are in line with evidence from studies conducted between 1979 and 2014, which report a high incidence among LTCF residents.^28^

COVID-19 was the third most reported type of HAI. Mild or moderate cases of COVID-19 were frequent among LTCF residents, but cases of severe COVID-19 (characterised by the need for oxygen therapy because of shortness of breath and/or an oxygen saturation level less than 92%) were much less frequent. This result reflects the stage of the COVID-19 pandemic during our study, when there was widespread circulation of omicron SARS-CoV-2 variants in Europe, which cause a milder form of disease. Extensive SARS-CoV-2 vaccination campaigns could also have had an effect on the incidence of severe COVID-19,^29^, ^30^ although we did not explore this factor in depth in this study.

The results for HAI-related adverse outcomes indicate that HAIs pose an increased risk of hospitalisation and death to LTCF residents in European countries. Deaths with HAIs identified as the main or secondary cause accounted for more than 20% of total deaths recorded during the study, with RTIs, UTIs, and COVID-19 mainly responsible. The incidence rates of HAI-related hospitalisations and deaths were 0·09 and 0·14 per 1000 resident-days, respectively, in this study; variable results have been reported elsewhere.^25^ As already noted, this variability might depend on several factors, including differences in the characteristics of the resident population, study period, and methodology used. Moreover, as expected, hospitalisations and, especially, deaths recorded 30 days after the onset of HAI were more frequent than those recorded at 7 days. According to the definitions of HAI-related hospitalisations and deaths used in this study, the 30-day estimates were likely to have included some deaths with causes other than HAIs, especially deaths of study participants for whom similar factors increased both the risk of acquiring an HAI and of dying. Therefore, adjustment for age and comorbidity remains relevant when discussing hospitalisations and mortality associated with HAIs in LTCF residents, particularly in LTCFs providing end-of-life care.

This study has some limitations. First, due to the longitudinal nature of the study and the need for the consistent presence of trained staff (which cannot always be guaranteed), we cannot exclude the possibility that logistical problems or staff shortages introduced some variability into implementation of the survey across the participating LTCFs. To increase the feasibility of longitudinal data collection in LTCFs—settings with limited resources—we encouraged countries to select LTCFs with existing HAI surveillance systems. Therefore, our selection of LTCFs might have been biased towards those with a high level of infection prevention and control activities and high awareness regarding the importance of HAIs. However, fewer than half of the LTCFs in this study reported an existing surveillance system, showing that LTCFs with less experience of infection surveillance were also included. Second, the different types of LTCFs included in the study resulted in some heterogeneity that reflects the varying characteristics of LTCF residents in Europe. Additionally, voluntary participation in the study resulted in different numbers of LTCFs included per country. Nevertheless, the characteristics of our overall study population were similar to those of populations reported in previous large European studies, which vary widely in terms of population characteristics because of diversity in national and regional long-term care systems.^2^ To account for heterogeneity between clusters, LTCFs, or countries, we used robust statistical methods and provided marginal estimates representative of the total sampled population. This approach increased the robustness of the estimated HAI incidence within the sampled LTCFs, allowing us to provide generalisable population estimates. Third, we did not consider the effect of infection prevention and control measures that were in place in each LTCF during the study, nor did we systematically assess the available local resources. Fourth, when attributing hospitalisations to HAIs, our assessments were done algorithmically and not directly. Finally, with the exception of COVID-19, we did not check the vaccination status or multidrug-resistant organism colonisation status of the study participants. These limitations must be considered when interpreting the results of this study. For example, infection prevention and control measures in LTCFs might decrease the incidence of some preventable HAIs, whereas high rates of multidrug-resistant organism colonisation could potentially lead to difficult-to-treat HAIs in the most clinically vulnerable residents.

The study also has several strengths, especially the use of standardised data collection tools with international definitions and the setting up of a specific data collection protocol with an existing network of nominated ECDC national focal points for HAIs in EU and EEA countries. The use of these methods resulted in a good level of participation by a wide range of LTCFs during a period in 2022–23 made challenging by the COVID-19 pandemic, during which almost all participating countries and LTCFs were able to complete the full 12 months of follow-up.

In conclusion, our findings show that HAIs, especially RTIs and UTIs, are a serious health issue for LTCF residents in the EU and EEA. LTCF residents, who are often older and frail, and frequently have multiple and severe underlying health conditions, are at increased risk of HAI-related adverse outcomes. These results suggest that future research to estimate the wider burden of HAIs and analyse risk factors for HAIs for the design of targeted local interventions in European LTCFs remains important.

### Contributors

### Data sharing

Pseudonymised case-based data or aggregate data are available on request of data for research purposes to the ECDC (https://www.ecdc.europa.eu/en/publications-data/request-tessy-data-research). Following approval of the data request, the relevant data can be shared through a secure online platform. The data can be made available for a minimum of 10 years from the end of the study.

## Declaration of interests

We declare no competing interests.
