# Supplementary material for: Incidence of health-care-associated infections in long-term care facilities in nine European countries: a 12-month, prospective, longitudinal cohort study
Source: Lancet Infect Dis. 2025 Nov;25(11):1199–207. doi: 10.1016/S1473-3099(25)00217-8 (PMC12547195; doi:10.1016/S1473-3099(25)00217-8)
Supplement: Supplementary appendix [file mmc1.pdf]

THE LANCET
Infectious Diseases

Supplementary appendix

This appendix formed part of the original submission and has been peer reviewed. We post it as supplied by the authors.

Supplement to: Ricchizzi E, Sasdelli E, Leucci AC, et al. Incidence of health-care-associated infections in long-term care facilities in nine European countries: a 12-month, prospective, longitudinal cohort study. *Lancet Infect Dis* 2025; published online June 16. https://doi.org/10.1016/S1473-3099(25)00217-8.

Supplementary materials

Table of contents

**Start and end date by country**	Pag. 1
**Ethical approval country list**	Pag. 1
**Table 1**. Questionnaires for data collection	Pag. 2
**Table 2**. Percentages of comorbidities among LTCF residents according to Charlson's index, by country.	Pag. 7
**Table 3**. Number, percentage, ratio and rate of HAIs, by type of HAI	Pag. 8
**Table 4**. Crude number, percentage, ratio and rate of HAIs, by type of HAI and country	Pag. 9
**Table 5**. Number, percentage, ratio and rate of hospitalisation for HAI, by type of HAI	Pag. 10
**Table 6**. Number, percentage, ratio and rate of hospitalisation, by type of HAI and country	Pag. 11
**Table 7**. Number, percentage, ratio and rate of death related to HAI, by type of HAI	Pag. 12
**Table 8**. Number, percentage, ratio and rate of death related to HAI, by type of HAI and country	Pag. 13
**H4LS study protocol**	Pag. 14

Start and end date of by country

Country's start date (as the date of the first LTCF started the data collection) and end date (as the date of the last LTCF ended the data collection): **Belgium** from 15/03/2022 to 14/03/2023; **Spain** from 17/01/2022 to 17/01/2023; **Finland** from 04/05/2022 to 03/05/2023; **France** from 01/01/2022 to 03/01/2023; **Italy** from 15/01/2022 to 12/05/2023; **Lithuania** from 17/01/2022 to 17/01/2023; **Luxembourg** from 15/03/2022 to 14/03/2023; **The Netherlands** from 01/01/2022 to 31/12/2022; **Poland** from 01/02/2022 to 31/01/2023.

Ethical approval country list

Belgium – Ref. Katrien Latour - an ethics committee and the Belgian Information Security Committee

Finland – Ref. Lehtinen Jaana-Marija – not required

France – Ref. Daniau Côme **-** not required

Greece – Ref. Kassiani Mellou - the Ethics Committee of the Greek National Public Health Organization

Italy – Ref. Enrico Ricchizzi for Ethical Commitee Area Vasta Emilia Nord (CET AVEN), Area Vasta Emilia Centro (CET AVEC) and Area Vasta Romagna (CEROM) and Costanza Vicentini for Ethical Committee of Turin University

Lithuania – Ref. Auguste Salnaite - not required

Luxembourg – Ref. Murielle Weydert - the National Research Ethics Committee of Luxembourg (*Comité National d'Éthique de Recherche – CNER*)

Netherlands – Ref. Kati Halonen – not required

Poland – Ref. Anna Różańska - The Bioethics Committee of the Jagiellonian University in Krakow approved the study under approval number KBET (1072.6120.247.2022)

Spain – Ref. Pilar Gallego Berciano - not required

Table 1. Questionnaires for data collection

Surveillance of healthcare-associated infections and antimicrobial use in European long-term care facilities (HALT)

One-year incidence of infections in long-term care residents: a prospective study

Institution Questionnaire

LTCFs questionnaire

Country		
LTCF Id		*Unique LTCF identification code given by the National coordinator*

LTCF characteristics

**Size (total beds)**			*Total number of beds in the facility at the beginning of the study*
**Type of LTCF**	❑ General Nursing home<br>❑ Mixed type facility	❑ Residential home<br>❑ Other	*Total number of beds in the facility at the beginning of the study*

**Are medical activities in the facility coordinated by a coordinating medical physician (CP)?**	❑ No, there is no internal or external coordination of the medical activity<br>❑ Yes, there is a physician from inside the facility (internal) who coordinates the medical activities<br>❑ Yes, there is a physician from outside the facility (external) who coordinates the medical activities<br>❑ Yes, there is both a physician from inside and outside the facility (internal and external) who coordinates the medical activities	*The Coordinating physician (CP) is a medical doctor in charge of the coordination of medical activities and standardisation of practices/policies in the facility.*
**Is a surveillance programme of healthcare-associated infections in place in the facility?**	❑ No<br>❑ Yes	*The LTCF perform a surveillance of infections on a regular basis, for example (there is an annual summary report of the number of urinary tract infections, respiratory tract infections, etc…).*
**Are laboratory tests routinely performed to diagnose an infection?**	❑ No<br>❑ Yes	*In the LTCF, laboratory test (e.g. microbiological exams) are routinely used to support the diagnosis of an infection. Note: the use of laboratory tests might depend on the infection type or on the physician attitude, please report the general practice for the most frequent infections.*

Survey dates

**From:**		*The date of the first day of data collection*
**To:**		*The date of the last day of data collection*

Study feasibility & workload

At the end of the survey, please report

**Total hours spent for the survey**	

Optional, please, specify the function, within the facility, of each data collector and the total hours spent for data collection

	Function	***Total hours for data collection***
**Data collector 1**	❑ Coordinating physician ❑ Infection control doctor ❑ Infection control nurse<br>❑ Medical doctor/general practitioner ❑ Head nurse ❑ Registered nurse<br>❑ Nursing assistant ❑ Other	
**Data collector 2**	❑ Coordinating physician ❑ Infection control doctor ❑ Infection control nurse<br>❑ Medical doctor/general practitioner ❑ Head nurse ❑ Registered nurse<br>❑ Nursing assistant ❑ Other	

**Data collector 3**	❑ Coordinating physician ❑ Infection control doctor ❑ Infection control nurse<br>❑ Medical doctor/general practitioner ❑ Head nurse ❑ Registered nurse<br>❑ Nursing assistant ❑ Other	
**Data collector 4**	❑ Coordinating physician ❑ Infection control doctor ❑ Infection control nurse<br>❑ Medical doctor/general practitioner ❑ Head nurse ❑ Registered nurse<br>❑ Nursing assistant ❑ Other	
**Data collector 5**	❑ Coordinating physician ❑ Infection control doctor ❑ Infection control nurse<br>❑ Medical doctor/general practitioner ❑ Head nurse ❑ Registered nurse<br>❑ Nursing assistant ❑ Other	

Comments

Please, report any comment

Max 800 characters

Surveillance of healthcare-associated infections and antimicrobial use in European long-term care facilities (HALT)

One-year incidence of infections in long-term care residents: a prospective study

Resident Questionnaire

Resident identifiers

Resident Name			
Resident Surname			
ResidentID			
Gender	❑ Male	❑ Female	❑ Unknown
Year of Birth	*yyyy*		
Date of admission in the LTCF	*dd/mm/yyyy*	________/________/________	

Resident's risk factors

**Disoriented**	❑ No	❑ Mild	❑ Moderate	❑ Severe	❑ Unknown
**Mobility**	❑ Ambulant	❑ Wheelchair	❑ Bedridden	❑ Unknown	
**Incontinence**	❑ Yes ❑ No	❑ Unknown			
**Urinary Catheter**	❑ Yes ❑ No	❑ Unknown	**Vascular Catheter**	❑ Yes ❑ No	❑ Unknown

Comorbidities

**Myocardial infarction**	❑ Yes	❑ No	***Decompensatio cordis***	❑ Yes	❑ No
**Peripheral arterial disease**	❑ Yes	❑ No	**Cerebrovascular disease**	❑ Yes	❑ No
**Dementia**	❑ Yes	❑ No	**Chronic lung disease**	❑ Yes	❑ No
**Peptic ulcer**	❑ Yes	❑ No	**Liver disorder**	❑No ❑ Mild ❑ Moderate/Severe	
**Diabetes**	❑No ❑ Mild ❑ Moderate/Severe		**Hemiplegia**	❑ Yes	❑ No
**Kidney disorders**	❑ Yes	❑ No	**Chronic urinary disorders**	❑ Yes	❑ No
**Malignancies**	❑ Yes	❑ No	**Leukaemia**	❑ Yes	❑ No
**Lymphoma**	❑ Yes	❑ No	**Metastasis**	❑ Yes	❑ No
**Systemic disease**	❑ Yes	❑ No			

COVID-19

History of infection				
**Previous COVID-19 episode**	❑ Yes	❑ No	❑ Unknown	
**Date of the previous COVID-19 episode**	*dd/mm/yyyy*	________ /________/________________		
**Disease severity of previous COVID-19 episode**	❑ asymptomatic	❑ Mild	❑ Moderate	❑ Severe
**More than one episode**	❑ Yes	❑ No	❑ Unknown	
Vaccination				

Pre-follow-up *(these is the status of the resident at the beginning of the follow-up)*

**COVID-19 vaccine offered**	❑ Yes	❑ No	❑ Unknown		
**Contraindication for the COVID-19 vaccine**	❑ Yes	❑ No	❑ Unknown		
**First dose of COVID-19 vaccine**	❑ Yes	❑ No	❑ Unknown	**Brand name of first dose vaccine received**	________________________________

**Date of the first dose**	________/________/___________	**Mode of vaccine ascertainment**	❑ not documented ❑ self-report ❑ vaccination card ❑ vaccination registry ❑ other

**Second dose of COVID-19 vaccine**	❑ Yes ❑ No ❑ Unknown	**Brand name of the second dose vaccine**	
**Date of the second dose**		**Mode of vaccine ascertainment of the second dose**	❑ not documented ❑ self-report ❑ vaccination card ❑ vaccination registry ❑ other

**Third dose of COVID-19 vaccine**	❑ Yes ❑ No ❑ Unknown	**Brand name of the third dose vaccine**	
**Date of the third dose**		**Mode of vaccine ascertainment of the third dose**	❑ not documented ❑ self-report ❑ vaccination card ❑ vaccination registry ❑ other

Post-follow-up *(this is the status of the resident at the end of the follow-up)*		
**COVID-19 vaccine offered**	❑ Yes ❑ No ❑ Unknown	
**Contraindication for the COVID-19 vaccine**	❑ Yes ❑ No ❑ Unknown	

**First dose of COVID-19 vaccine**	❑ Yes ❑ No ❑ Unknown	**Brand name of first dose vaccine received**	______________________________
**Date of the first dose**	________/________/___________	**Mode of vaccine ascertainment**	❑ not documented ❑ self-report ❑ vaccination card ❑ vaccination registry ❑ other

**Second dose of COVID-19 vaccine**	❑ Yes ❑ No ❑ Unknown	**Brand name of the second dose vaccine**	
**Date of the second dose**		**Mode of vaccine ascertainment of the second dose**	❑ not documented ❑ self-report ❑ vaccination card ❑ vaccination registry ❑ other

**Third dose of COVID-19 vaccine**	❑ Yes ❑ No ❑ Unknown	**Brand name of the third dose vaccine**	
**Date of the third dose**		**Mode of vaccine ascertainment of the third dose**	❑ not documented ❑ self-report ❑ vaccination card ❑ vaccination registry ❑ other

End of the Follow-up

**Date of the end of the follow-up**	___________________/ ___________________/ ___________________
**Status at the end of the follow up**	❑ Resident is alive, in the current LTCF<br>❑ Resident is alive, temporary discharged (no hospital)<br>❑ Resident is deceased<br>❑ Resident is alive, at the hospital<br>❑ Resident is alive, permanently discharged (no hospital)<br>❑ Resident status is unknown

Surveillance of healthcare-associated infections and antimicrobial use in European long-term care facilities (HALT)

One-year incidence of infections in long-term care residents: a prospective study

Resident Questionnaire

Resident identifiers

ResidentID	

Temporary discharges

Date of temporary discharge	Date of re-admission in the current LTCF	Place where discharged	Reason for the hospitalisation
		❑ Home ❑hospital urgent ❑ hospital planned ❑ other LTCF ❑ Unknown	❑ Medical ❑ Surgical ❑ Diagnostic procedures

Surveillance of healthcare-associated infections and antimicrobial use in European long-term care facilities (HALT)

One-year incidence of infections in long-term care residents: a prospective study

HAI Questionnaire

Resident identifiers

ResidentID	

HAI

Infection site	
If OTHER, please specify	
Start date	
End date	
Infection outcome	❑ Alive<br>❑ Death as part of the causal sequence<br>❑ Death, no contribution<br>❑ Unknown status or not verified<br>❑ Death, sole cause<br>❑ Death as contributory cause<br>❑ Unknown or not verified
Infection diagnosed in hospital	❑ Yes ❑ No ❑ Unknown

Microorganisms

Name of the **first** mm.o isolated		S/I/R
Tested antimicrobials and resistance		
Name of the **second** mm.o isolated		S/I/R
Tested antimicrobials and resistance		
Name of the **third** mm.o isolated		S/I/R
Tested antimicrobials and resistance		

Comments & notes

Report any comment and/or notes – this is for internal use, do not report in the electronic data collection tools

Table 2. Percentages of comorbidities among LTCF residents according to Charlson's index, by country.

Comorbidity	Belgium*	Finland	France	Italy	Lithuania	Luxembourg	Netherlands	Poland	Spain	Total**
**Myocardial infarction**	-	4·94	9·12	7·59	1·90	0·65	7·63	16·36	4·56	7·11
**Decompensation cordis**	-	19·77	20·51	18·48	17·66	0·65	12·36	48·7	17·11	20·26
**Peripheral arterial disease**	-	7·29	8·55	13·16	13·59	1·31	5·82	75·84	7·6	15·49
**Cerebrovascular disease**	-	31·98	22·51	34·18	19·57	7·19	31·64	51·3	14·07	27·01
**Dementia**	-	80·23	73·08	41·77	15·22	20·92	50·91	71·38	83·27	57·53
**Chronic lung disease**	-	11·92	10·83	16·2	4·35	15·69	5·82	6·69	9·51	10·11
**Peptic ulcer**	-	1·74	6·84	2·78	2·17	7·19	1·45	8·55	3·04	4·30
**Mild liver disorder**	-	0·29	4·13	3·04	1·90	1·96	1·09	4·09	4·56	2·53
**Moderate/severe liver disorder**	-	0	1	0·51	1·63	0	0·36	3·72	0·38	0·98
**Hemiplegia**	-	6·69	3·85	6·33	4·62	2·61	13·09	13·75	2·66	6·36
**Kidney disorder**	-	12·21	15·1	9·62	1·63	4·58	5·45	17·1	10·65	10·40
**Chronic urinary disorder**	-	28·78	15·38	15·19	3·8	24·84	6·18	11·52	18·25	14·99
**Malignancy**	-	7·85	9·69	8·61	2·72	4·58	9·09	5·58	12·17	7·87
**Leukaemi**a	-	0	1	0·76	0·54	1·96	1·82	0·37	1·52	0·90
**Lymphoma**	-	0·87	0·85	1·77	0·27	0·65	0·36	0	0·38	0·69
**Metastasis**	-	0·87	1	1·77	0·82	0	2·91	0	0·38	1·05
**Systemic disease**	-	11·34	2·85	10·63	2·17	0·65	18·55	5·58	5·7	6·90
**Mild diabetes**	-	22·97	14·96	16·2	3·8	9·15	16·73	6·69	19·01	14·08
**Moderate/severe diabetes**	-	4·94	6·55	7·09	2·45	0·65	4·00	21·93	3·04	6·46

* Data not available; ** Values computed excluding missing observations

Table 3. Number, percentage, ratio and rate of HAIs, by type of HAI

Type of HAI	N	Crude %	Estimated %(a)	Lower 95% CI	Upper 95% CI	Crude Ratio	Estimated Ratio(b)	Lower 95% CI	Upper 95% CI	Crude Rate	Estimated Rate(b)	Lower 95% CI	Upper 95% CI
**Total**	3763	100				124·19	124·12	118·59	129·93	3·93	1·76	0·94	3·31
**RTI**	1080	28·70	28·86	27·31	30·46	35·65	35·61	33·19	38·21	1·13	1·14	1·01	1·33
Other lower respiratory tract infection	394	10·47	10·65	9·60	11·80	13·00	11·92	10·62	13·38	0·41	0·35	0·27	0·47
Pneumonia	279	7·41	7·29	6·38	8·33	9·21	7·87	6·81	9·08	0·29	0·27	0·11	0·67
Common cold	273	7·25	7·25	6·42	8·17	9·01	7·81	6·86	8·89	0·28	0·31	0·22	0·42
Seasonal influenza	134	3·56	3·57	3·02	4·23	4·42	3·71	3·11	4·42	0·14	0·16	0·09	0·29
**UTI**	743	19·74	18·70	17·24	20·26	24·52	24·49	22·19	27·03	0·78	0·76	0·60	0·95
Confirmed	434	11·56	10·68	9·59	11·89	14·36	11·96	10·61	13·49	0·45	0·49	0·37	0·65
Probable	9	8·18	8·19	7·25	9·23	10·17	8·92	7·82	10·17	0·32	0·22	0·15	0·33
**COVID-19**	687	18·26	17·59	16·46	18·78	22·67	22·67	21·16	24·29	0·72	0·55	0·39	0·79
Mild/Moderate	615	16·34	15·95	14·86	17·12	20·30	18·98	17·45	20·65	0·64	0·55	0·39	0·77
Severe	72	1·91	1·92	1·52	2·41	2·38	1·95	1·55	2·47	0·08	0·06	0·04	0·08
**Skin and soft tissue**	582	15·47	14·30	13·05	15·66	19·21	19·21	17·22	21·43	0·61	0·59	0·39	0·88
Fungal	304	8·08	6·82	5·94	7·81	10·03	7·32	6·31	8·48	0·32	0·33	0·19	0·57
Cellulitis/soft tissue/wound infection	243	6·46	6·39	5·57	7·31	8·02	6·82	5·90	7·89	0·25	0·24	0·16	0·37
Herpes	27	0·72	0·72	0·49	1·06	0·89	0·73	0·49	1·08	0·03	0·03	0·02	0·04
Scabies	8	0·21	0·21	0·11	0·42	0·26	0·21	0·11	0·43	0·01	0·01	0·00	0·02
**GI**	262	6·96	6·96	6·17	7·84	8·65	8·65	7·61	9·83	0·27	0·23	0·13	0·39
Gastroenteritis	244	6·48	6·49	5·73	7·35	8·05	6·94	6·08	7·93	0·25	0·23	0·14	0·39
Clostridioides difficile infection	18	0·48	0·42	0·25	0·71	0·59	0·42	0·25	0·71	0·02	0·00	0·00	0·09
**Eye,ear,nose and mouth**	236	6·27	6·10	5·30	7·02	7·79	7·79	6·69	9·06	0·25	0·22	0·15	0·32
Conjunctivitis	154	4·09	4·11	3·45	4·89	5·08	4·29	3·58	5·14	0·16	0·15	0·10	0·22
Mouth infection	54	1·44	1·22	0·89	1·67	1·78	1·23	0·89	1·70	0·06	0·04	0·01	0·10
Ear infection	23	0·61	0·58	0·37	0·92	0·76	0·59	0·37	0·92	0·02	0·02	0·01	0·04
Sinusitis	5	0·13	0·13	0·06	0·32	0·17	0·13	0·06	0·32	0·01	0·00	0·00	0·02
**Other**	109	2·90	2·92	2·38	3·58	3·60	3·60	2·90	4·46	0·11	0·11	0·08	0·16
**Unexplained febrile episode**	33	0·88	0·92	0·63	1·34	1·09	1·09	0·75	1·59	0·03	0·04	0·02	0·07
**Bloodstream**	17	0·45	0·43	0·26	0·71	0·56	0·56	0·34	0·93	0·02	0·02	0·01	0·03
**Surgical site·**	14	0·37	0·37	0·21	0·64	0·46	0·46	0·26	0·81	0·01	0·01	0·01	0·03
Superficial incision	9	0·24	0·25	0·12	0·51	0·30	0·25	0·12	0·52	0·01	0·01	0·00	0·02
Organ/space	4	0·11	0·11	0·04	0·28	0·13	0·11	0·04	0·28	0·00	0·00	0·00	0·01
Deep incisional	1	0·03	0·03	0·00	0·19	0·03	0·03	0·00	0·19	0·00	0·00	0·00	0·01

Note: Results from only-intercept GEE models with exchangeable correlation structure: (a) binomial models adjusted for resident measures and clustering in LTCFs, N=3,763 HAIs; (b) Poisson models adjusted for clustering in LTCFs, N= 3,029 residents; CI = Confidence Intervals. RTI: respiratory tract infection; UTI: urinary tract infection; GI: gastrointestinal infection. 'Other' includes HAIs that did not meet any specific case definitions used in the study.

Table 4. Crude number, percentage, ratio and rate of HAIs, by type of HAI and country

Type of HAI	Belgium				Finland				France			
	Total*	%	Ratio	Rate	Total	%	Ratio	Rate	Total	%	Ratio	Rate
RTI	149	30·79	57·09	1·75	125	25·15	36·34	1·22	285	24·19	40·60	1·28
UTI	82	16·94	31·42	0·96	147	29·58	42·73	1·43	183	15·53	26·07	0·82
COVID-19	106	21·90	40·61	1·24	67	13·48	19·48	0·65	230	19·52	32·76	1·03
Skin and soft tissue	60	12·40	22·99	0·70	87	17·51	25·29	0·85	238	20·20	33·90	1·07
GI	40	8·26	15·33	0·47	17	3·42	4·94	0·17	107	9·08	15·24	0·48
Eye, ear, nose and mouth	19	3·93	7·28	0·22	26	5·23	7·56	0·25	75	6·37	10·68	0·34
Other	22	4·55	8·43	0·26	12	2·41	3·49	0·12	44	3·74	6·27	0·20
Unexplained febrile episode	1	0·21	0·38	0·01	13	2·62	3·78	0·13	7	0·59	1·00	0·03
Bloodstream	3	0·62	1·15	0·04	1	0·20	0·29	0·01	8	0·68	1·14	0·04
Surgical site	2	0·41	0·77	0·02	2	0·40	0·58	0·02	1	0·08	0·14	0·00
	Italy				Lithuania				Luxembourg			
	Total	%	Ratio	Rate	Total	%	Ratio	Rate	Total	%	Ratio	Rate
RTI	93	31·42	23·54	0·75	42	47·73	11·41	0·33	39	26·90	25·49	0·73
UTI	48	16·22	12·15	0·39	12	13·64	3·26	0·09	16	11·03	10·46	0·30
COVID-19	83	28·04	21·01	0·67	18	20·45	4·89	0·14	59	40·69	38·56	1·11
Skin and soft tissue	21	7·09	5·32	0·17	1	1·14	0·27	0·01	17	11·72	11·11	0·32
GI	22	7·43	5·57	0·18	2	2·27	0·54	0·02	1	0·69	0·65	0·02
Eye, ear, nose and mouth	17	5·74	4·30	0·14	5	5·68	1·36	0·04	8	5·52	5·23	0·15
Other	7	2·36	1·77	0·06	4	4·55	1·09	0·03	1	0·69	0·65	0·02
Unexplained febrile episode	4	1·35	1·01	0·03	4	4·55	1·09	0·03	0	0·00	0·00	0·00
Bloodstream	0	0·00	0·00	0·00	0	0·00	0·00	0·00	0	0·00	0·00	0·00
Surgical site	1	0·34	0·25	0·01	0	0·00	0·00	0·00	4	2·76	2·61	0·08
	Netherlands				Poland				Spain			
	Total	%	Ratio	Rate	Total	%	Ratio	Rate	Total	%	Ratio	Rate
RTI	64	21·40	23·27	0·73	201	48·32	74·72	2·52	82	22·91	31·18	0·99
UTI	84	28·09	30·55	0·30	87	20·91	32·34	1·09	84	23·46	31·94	1·02
COVID-19	81	27·09	29·45	1·11	16	3·85	5·95	0·20	27	7·54	10·27	0·33
Skin and soft tissue	54	18·06	19·64	0·32	28	6·73	10·41	0·35	76	21·23	28·90	0·92
GI	11	3·68	4·00	0·02	31	7·45	11·52	0·39	31	8·66	11·79	0·38
Eye, ear, nose and mouth	4	1·34	1·45	0·15	37	8·89	13·75	0·46	45	12·57	17·11	0·55
Other	1	0·33	0·36	0·02	10	2·40	3·72	0·13	8	2·23	3·04	0·10
Unexplained febrile episode	2	0·67	0·73	0·00	2	0·48	0·74	0·03	0	0·00	0·00	0·00
Bloodstream	0	0·00	0·00	0·00	4	0·96	1·49	0·05	1	0·28	0·38	0·01
Surgical site	0	0·00	0·00	0·08	0	0·00	0·00	0·00	4	1·12	1·52	0·05

*Number of HAIs; RTI: respiratory tract infection; UTI: urinary tract infection; GI: gastrointestinal infection. ‘Other’ includes HAIs that did not meet any specific case definitions used in the study.

Table 5. Number, percentage, ratio and rate of hospitalisation for HAI, by type of HAI

Type of HAI	N	Crude %	Estimated %[(a)]	Lower 95% CI	Upper 95% CI	Crude Ratio	Estimated Ratio[(b)]	Lower 95% CI	Upper 95% CI	Crude Rate	Estimated rate[(b)]	Lower 95% CI	Upper 95% CI
**Total**	160	4·28	4·33	3·90	5·45	5·32	5·02	4·10	7·62	0·13	0·09	0·05	0·21
**RTI**	63	1·67	1·75	1·37	2·23	2·08	2·12	1·32	3·21	0·01	0·02	0·01	0·09
Other lower respiratory tract infection	16	0·43	0·45	0·28	0·72	0·53	0·58	0·33	1·02	0·02	0·01	0·01	0·03
Pneumonia	37	0·98	1·01	0·73	1·38	1·22	1·13	0·71	1·81	0·03	0·01	0·00	0·06
Common cold	2	0·05	0·08	0·03	0·25	0·07	0·10	0·04	0·29	0·00	0·00	0·00	0·01
Seasonal influenza	8	0·21	0·21	0·11	0·42	0·26	0·33	0·09	1·25	0·01	0·00	0·00	0·03
**UTI**	32	0·85	0·89	0·66	1·26	1·06	1·14	0·81	1·67	0·03	0·02	0·01	0·05
Confirmed	23	0·61	0·63	0·42	0·96	0·76	0·80	0·56	1·13	0·03	0·02	0·01	0·04
Probable	9	0·24	0·26	0·12	0·49	0·30	0·34	0·17	0·74	0·01	0·00	0·00	0·02
**COVID-19**	27	0·72	0·81	0·57	1·20	0·89	1·09	0·70	1·77	0·03	0·02	0·01	0·04
Mild/Moderate	10	0·27	0·32	0·18	0·44	0·33	0·45	0·23	0·99	0·01	0·01	0·00	0·03
Severe	17	0·45	0·48	0·30	0·76	0·56	0·61	0·37	0·99	0·02	0·01	0·01	0·03
**Skin and soft tissue**	11	0·32	0·40	0·24	0·66	0·40	0·50	0·28	0·95	0·02	0·01	0·01	0·03
Fungal	1	0·03	0·08	0·03	0·25	0·03	0·10	0·04	0·29	0·00	0·00	0·00	0·01
Cellulitis/soft tissue/wound	10	0·29	0·32	0·18	0·56	0·36	0·42	0·22	0·79	0·01	0·01	0·00	0·02
**GI**	5	0·13	0·10	0·04	0·27	0·17	0·17	0·07	0·42	0·01	0·00	0·00	0·01
Gastroenteritis	2	0·05	0·05	0·01	0·21	0·07	0·07	0·02	0·25	0·00	0·00	0·00	0·01
Clostridioides difficile	3	0·08	0·04	0·01	0·17	0·10	0·10	0·03	0·39	0·00	0·00	0·00	0·01
**Eye, ear, nose and mouth**	3	0·00	0·13	0·06	0·32	0·00	0·17	0·06	0·46	0·01	0·00	0·00	0·01
Mouth infection	2	0·05	0·05	0·01	0·21	0·07	0·07	0·01	0·47	0·00	0·00	0·00	0·01
Ear infection	0	0·00	0·03	0·00	0·19	0·00	0·03	0·00	0·24	0·00	0·00	0·00	0·01
**Other infection**	9	0·00	0·24	0·13	0·46	0·00	0·30	0·16	0·57	0·01	0·01	0·00	0·02
**Unexplained febrile episode**	2	0·00	0·05	0·01	0·21	0·00	0·09	0·03	0·29	0·00	0·00	0·00	0·01
**Bloodstream**	6	0·00	0·16	0·07	0·35	0·00	0·18	0·07	0·45	0·01	0·00	0·00	0·01
**Surgical site**	2	0·00	0·05	0·01	0·21	0·00	0·07	0·02	0·25	0·00	0·00	0·00	0·01
Superficial incision	1	0·03	0·03	0·00	0·19	0·03	0·03	0·00	0·24	0·00	0·00	0·00	0·01
Deep incisional	1	0·03	0·03	0·00	0·19	0·03	0·04	0·01	0·25	0·00	0·00	0·00	0·01

Note: Results from only-intercept GEE models with exchangeable correlation structure: (a) binomial models adjusted for resident measures and clustering in LTCFs, N=3763 infections; (b) Poisson models adjusted for clustering in LTCFs, N= 3029 residents; CI = Confidence Intervals. RTI: respiratory tract infection; UTI: urinary tract infection; GI: gastrointestinal infection. ‘Other’ includes HAIs that did not meet any specific case definitions used in the study.

Table 6. Number, percentage, ratio and rate of hospitalisation, by type of HAI and country

**Type of HAI**	**Belgium**				**Finland**				**France**			
	**Total***	**%**	**Ratio**	**Rate**	**Total**	**%**	**Ratio**	**Rate**	**Total**	**%**	**Ratio**	**Rate**
**RTI**	16	3·31	6·15	0·08	3	0·60	0·87	0·01	15	1·27	2·14	0·06
**UTI**	5	1·03	1·92	0·01	1	0·20	0·29	0·00	10	0·85	1·42	0·05
**COVID-19**	2	0·41	0·77	0·01	0	0·00	0·00	0·00	5	0·42	0·71	0·00
**Skin and soft tissue**	3	0·83	1·54	0·03	0	0·00	0·00	0·00	4	0·34	0·57	0·03
**GI**	2	0·41	0·77	0·05	0	0·00	0·00	0·00	2	0·17	0·28	0·01
**Eye, ear, nose and mouth**	0	0·00	0·00	0·00	0	0·00	0·00	0·00	0	0·00	0·00	0·00
**Other**	1	0·21	0·38	0·00	0	0·00	0·00	0·00	4	0·34	0·57	0·00
**Unexplained febrile episode**	0	0·00	0·00	0·00	0	0·00	0·00	0·00	0	0·00	0·00	0·00
**Bloodstream**	2	0·41	0·77	0·01	1	0·20	0·29	0·00	0	0·00	0·00	0·00
**Surgical site**	0	0·00	0·00	0·00	0	0·00	0·00	0·00	0	0·00	0·00	0·00
	**Italy**				**Lithuania**				**Luxembourg**			
	**Total**	**%**	**Ratio**	**Rate**	**Total**	**%**	**Ratio**	**Rate**	**Total**	**%**	**Ratio**	**Rate**
**RTI**	7	1·45	1·77	0·02	2	0·40	0·54	0·01	3	0·25	1·96	0·02
**UTI**	6	1·24	1·52	0·02	0	0·00	0·00	0·00	0	0·00	0·00	0·00
**COVID-19**	10	2·07	2·53	0·00	0	0·00	0·00	0·00	3	0·25	1·96	0·02
**Skin and soft tissue**	0	0·00	0·00	0·00	0	0·00	0·00	0·00	0	0·00	0·00	0·00
**GI**	0	0·00	0·00	0·00	0	0·00	0·00	0·00	0	0·00	0·00	0·00
**Eye, ear, nose and mouth**	1	0·21	0·25	0·01	0	0·00	0·00	0·00	0	0·00	0·00	0·00
**Other**	1	0·21	0·25	0·00	0	0·00	0·00	0·00	0	0·00	0·00	0·00
**Unexplained febrile episode**	0	0·00	0·00	0·00	0	0·00	0·00	0·00	0	0·00	0·00	0·00
**Bloodstream**	0	0·00	0·00	0·00	0	0·00	0·00	0·00	0	0·00	0·00	0·00
**Surgical site**	0	0·00	0·00	0·00	0	0·00	0·00	0·00	1	0·08	0·65	0·00
	**Netherlands**				**Poland**				**Spain**			
	**Total**	**%**	**Ratio**	**Rate**	**Total**	**%**	**Ratio**	**Rate**	**Total**	**%**	**Ratio**	**Rate**
**RTI**	0	0·00	0·00	0·00	10	2·01	3·72	0·05	7	0·59	2·66	0·06
**UTI**	4	0·83	1·45	0·03	2	0·40	0·74	0·03	4	0·34	1·52	0·04
**COVID-19**	5	1·03	1·82	0·03	0	0·00	0·00	0·00	2	0·17	0·76	0·01
**Skin and soft tissue**	2	0·41	0·73	0·00	0	0·00	0·00	0·00	2	0·17	0·76	0·01
**GI**	0	0·00	0·00	0·00	1	0·20	0·37	0·01	0	0·00	0·00	0·00
**Eye, ear, nose and mouth**	1	0·21	0·36	0·01	1	0·20	0·37	0·01	0	0·00	0·00	0·00
**Other**	0	0·00	0·00	0·00	1	0·20	0·37	0·00	2	0·17	0·76	0·01
**Unexplained febrile episode**	1	0·21	0·36	0·01	1	0·20	0·37	5	0	0·00	0·00	0·00
**Bloodstream**	0	0·00	0·00	0·00	3	0·60	1·12	0·04	0	0·00	0·00	0·00
**Surgical site**	0	0·00	0·00	0·00	0	0·00	0·00	0·00	1	0·08	0·38	0·00

*Number of HAIs; RTI: respiratory tract infection; UTI: urinary tract infection; GI: gastrointestinal infection· 'Other' includes HAIs that did not meet any specific case definitions used in the study.

Table 7. Number, percentage, ratio and rate of death related to HAI, by type of HAI

Type of HAI	N[1]	Crude %	Estimated %[a]	Lower 95% CI	Upper 95% CI	Crude Ratio	Estimated Ratio[b]	Lower 95% CI	Upper 95% CI	Crude Rate	Estimated Rate[b]	Lower 95% CI	Upper 95% CI
**Total**	154	4·09	4·50	2·45	4·79	4·69[1]	4·01	2·85	5·63	0·15	0·14	0·08	0·23
**RTI**	85	2·26	2·25	1·82	2·77	2·81	2·25	1·47	3·43	0·09	0·09	0·05	0·15
Other lower respiratory tract	39	1·04	2·45	1·93	3·12	1·29	1·38	0·82	2·31	0·01	0·01	0·00	0·06
Pneumonia	41	1·09	2·83	2·28	3·50	1·35	0·66	0·28	1·55	0·04	0·04	0·01	0·13
Common cold	1	0·03	1·14	0·83	1·56	0·03	0·03	0·01	0·23	0·00	0·00	0·00	0·01
Seasonal influenza	4	0·11	0·60	0·39	0·91	0·13	0·13	0·05	0·34	0·00	0·00	0·00	0·01
**UTI**	16	0·43	0·43	0·26	0·69	0·53	0·52	0·32	0·86	0·02	0·02	0·01	0·03
Confirmed	10	0·27	2·33	1·84	2·96	0·33	0·30	0·17	0·53	0·01	0·01	0·01	0·02
Probable	6	0·16	1·80	1·37	2·36	0·20	0·15	0·06	0·39	0·00	0·00	0·00	0·01
**COVID-19**	18	0·48	0·48	0·30	0·76	0·59	0·58	0·29	1·18	0·02	0·02	0·01	0·04
Mild/Moderate	5	0·13	2·90	2·40	3·50	0·17	0·19	0·06	0·59	0·00	0·00	0·00	0·01
Severe	13	0·35	0·88	0·63	1·25	0·43	0·39	0·17	0·91	0·01	0·01	0·01	0·03
**Skin and soft tissue·**	6	0·16	0·16	0·07	0·36	0·20	0·20	0·10	0·39	0·01	0·01	0·00	0·01
Cellulitis/soft tissue/wound	6	0·16	1·52	1·13	2·03	0·20	0·20	0·10	0·39	0·01	0·01	0·00	0·01
**GI**	8	0·21	0·21	0·11	0·42	0·26	0·25	0·10	0·63	0·01	0·00	0·00	0·03
Gastroenteritis	4	0·11	1·22	0·91	1·64	0·13	0·16	0·04	0·57	0·01	0·01	0·00	0·02
Clostridioides difficile	4	0·11	0·23	0·12	0·45	0·13	0·08	0·02	0·31	0·00	0·00	0·00	0·02
**Eye, ear, nose and mouth**	2	0·05	0·05	0·01	0·21	0·07	0·04	0·01	0·25	0·00	0·00	0·00	0·01
**Other infection**	7	0·19	0·19	0·09	0·39	0·23	0·24	0·13	0·46	0·01	0·01	0·00	0·02
**Unexplained febrile episode**	8	0·21	0·21	0·11	0·43	0·26	0·32	0·11	0·99	0·01	0·01	0·00	0·02
**Bloodstream**	4	0·11	0·11	0·04	0·28	0·13	0·13	0·06	0·32	0·00	0·00	0·00	0·01
**Surgical site**	0	-	-	-	-	·	·	·	·	-	-	-	-

Note: Results from only-intercept binomial GEE models with exchangeable correlation structure: (a) adjusted estimates for resident measures and clustering in LTCFs, N=3,763 HAIs; (b) adjusted estimates for clustering in LTCFs, N= 3,029 residents; (1) 142 residents who died because of the infection were included. The sum of the following rows is equal to the 154 infections that resulted in death; this difference is due to multiple infections contributing to death; CI = Confidence Intervals; RTI: respiratory tract infection; UTI: urinary tract infection; GI: gastrointestinal infection. 'Other' includes HAIs that did not meet any specific case definitions used in the study.

Table 8. Number, percentage, ratio and rate of death related to HAI, by type of HAI and country

Type of HAI	Belgium				Finland				France			
	Total*	%	Ratio	Rate	Total*	%	Ratio	Rate	Total*	%	Ratio	Rate
RTI	3	0·62	1·15	0·04	5	1·01	1·45	0·05	28	2·38	3·99	0·13
UTI	1	0·21	0·38	0·01	0	0·00	0·00	0·00	8	0·68	1·14	0·04
COVID-19	2	0·41	0·77	0·02	0	0·00	0·00	0·00	7	0·59	1·00	0·03
Skin and soft tissue	1	0·21	0·38	0·01	0	0·00	0·00	0·00	2	0·17	0·28	0·01
GI	0	0·00	0·00	0·00	0	0·00	0·00	0·00	2	0·17	0·28	0·01
Eye, ear, nose and mouth	0	0·00	0·00	0·00	0	0·00	0·00	0·00	0	0·00	0·00	0·00
Other	3	0·62	1·15	0·04	0	0·00	0·00	0·00	1	0·08	0·14	0·00
Unexplained febrile episode	1	0·21	0·38	0·01	1	0·20	0·29	0·01	0	0·00	0·00	0·00
Bloodstream	0	0·00	0·00	0·00	0	0·00	0·00	0·00	3	0·25	0·43	0·01
Surgical site	0	0·00	0·00	0·00	0	0·00	0·00	0·00	0	0·00	0·00	0·00
	Italy				Lithuania				Luxembourg			
	Total*	%	Ratio	Rate	Total*	%	Ratio	Rate	Total*	%	Ratio	Rate
RTI	2	0·68	0·51	0·02	1	1·14	0·27	0·01	0	0·00	0·00	0·00
UTI	1	0·34	0·25	0·01	0	0·00	0·00	0·00	0	0·00	0·00	0·00
COVID-19	0	0·00	0·00	0·00	0	0·00	0·00	0·00	0	0·00	0·00	0·00
Skin and soft tissue	1	0·34	0·25	0·01	0	0·00	0·00	0·00	0	0·00	0·00	0·00
GI	2	0·68	0·51	0·02	0	0·00	0·00	0·00	0	0·00	0·00	0·00
Eye, ear, nose and mouth	0	0·00	0·00	0·00	0	0·00	0·00	0·00	0	0·00	0·00	0·00
Other	1	0·34	0·25	0·01	0	0·00	0·00	0·00	0	0·00	0·00	0·00
Unexplained febrile episode	0	0·00	0·00	0·00	4	4·55	1·09	0·03	0	0·00	0·00	0·00
Bloodstream	0	0·00	0·00	0·00	0	0·00	0·00	0·00	0	0·00	0·00	0·00
Surgical site	0	0·00	0·00	0·00	0	0·00	0·00	0·00	0	0·00	0·00	0·00
	Netherlands				Poland				Spain			
	Total*	%	Ratio	Rate	Total*	%	Ratio	Rate	Total*	%	Ratio	Rate
RTI	11	3·68	4·00	0·14	27	6·49	10·04	0·34	8	2·23	3·04	0·10
UTI	1	0·33	0·36	0·01	4	0·96	1·49	0·05	1	0·28	0·38	0·01
COVID-19	8	2·68	2·91	0·10	0	0·00	0·00	0·00	1	0·28	0·38	0·01
Skin and soft tissue	1	0·33	0·36	0·01	1	0·24	0·37	0·01	0	0·00	0·00	0·00
GI	1	0·33	0·36	0·01	3	0·72	1·12	0·04	0	0·00	0·00	0·00
Eye, ear, nose and mouth	0	0·00	0·00	0·00	0	0·00	0·00	0·00	2	0·56	0·76	0·01
Other	0	0·00	0·00	0·00	1	0·24	0·37	0·01	1	0·28	0·38	0·01
Unexplained febrile episode	1	0·33	0·36	0·01	1	0·24	0·37	0·01	0	0·00	0·00	0·00
Bloodstream	0	0·00	0·00	0·00	1	0·24	0·37	0·01	0	0·00	0·00	0·00
Surgical site	0	0·00	0·00	0·00	0	0·00	0·00	0·00	0	0·00	0·00	0·00

*Number of HAIs; RTI: respiratory tract infection; UTI: urinary tract infection; GI: gastrointestinal infection. ‘Other’ includes HAIs that did not meet any specific case definitions used in the study.

One-year incidence of infections in long-term care facility residents: a longitudinal study (H4LS)

Internal Study Protocol version 1.6

Contents

Abbreviations 16
1 Introduction 17
2 Objectives 17
3 Methods 18
3·1 Design 18
3·2 Survey population 18
3·3 Data collection tools 19
3·4 Data collectors 28
4· Training 29
5· Role of the national survey coordinator 29
6· Data delivery 29
6·1 Electronic data collection tools 29
6·2 Deadline for data delivery 29
6·3 Data analysis and feedback 30
6·4 Data ownership 30
7· Ethical considerations 30
8· References 30
Annex 1· Institutional questionnaire 32
Annex 2· Resident questionnaire 34
Annex 3· Infection questionnaire 37
Annex 4· Case definitions of infections 37
Annex 5· Code list with microorganisms 45

Abbreviations

AMR	Antimicrobial resistance
DALY	Disability-adjusted life years
EEA	European Economic Area
ESAC-NH	European Surveillance of Antimicrobial Consumption in Nursing Homes
EU	European Union
GP	General practitioner
HAI	Healthcare-associated infection
HALT	Healthcare-associated infections in long-term care facilities project, 2010
HALT-2	Healthcare-associated infections and antimicrobial use in long-term care facilities project, 2013
HALT-3	Healthcare-associated infections and antimicrobial use in long-term care facilities project, 2016 – 2017
IPC	Infection prevention and control
IPSE	Improving Patient Safety in Europe project
LTCF	Long-term care facility
NSC	National survey coordinator
PPS	Point prevalence survey
RTI	Respiratory tract infection
UTI	Urinary tract infection

1 Introduction

Since 2008, the European Centre for Disease Prevention and Control (ECDC) is organising repeated point prevalence surveys (PPSs) of healthcare-associated infections (HAIs) and antimicrobial use in European long-term care facilities (LTCFs) through the Healthcare-Associated Infections and Antimicrobial Use in Long-Term Care Facilities (HALT) projects. Based on the HAI prevalence results of the first HALT PPS (HALT (2010))[1] and the second HALT PPS (HALT-2 (2013))[2], the total annual burden of HAIs in European LTCFs was estimated at respectively 4.3 million infection episodes (of which 2.6 million matched the case definition) in 2010 [3] and 4.2 million infection episodes in 2013 [2]. These estimates were based on the HAI prevalence, the estimated number of LTCF beds in Europe collected at the national level (approximately 3.6 million LTCF beds in 63 000 LTCFs in 2013), a bed occupancy of 95% [4] and the assumption that the duration of a HAI is on average 10 days. In the protocol of the third European PPS in LTCFs (HALT-3 survey, 2016-2017[5]) the date of onset of infection was added to refine the estimation of the latter parameter (duration of infection) using estimates from the survey for the different HAI types.

However, from the PPS data alone, it is not possible to estimate the number of LTCF residents in whom the estimated annual number of HAI episodes occur. This information is very important in view of the estimation of the total burden of HAIs in European LTCFs, expressed in disability-adjusted life years (DALYs) as was recently done for acute care hospitals [6]. Only few HAI incidence studies were published for the LTCF setting, and at our knowledge none of them looked at the incidence of infections at the resident level. In addition, another crucial information which is missing for the burden estimation at the EU level is an estimation of the attributable mortality of infections specifically for LTCF-associated infections, for which case definitions differ from the HAI case definitions used in the acute care setting.

2 Objectives

The objectives of the current longitudinal study are:

- To estimate the percentage of LTCF residents who acquire no, one, two, three or more infections during a one-year period in an average LTCF in Europe, overall and stratified by main risk factors of infections
- To estimate the attributable mortality of LTCF-associated infections
- To allow the estimation of the incidence of LTCF-associated infections in a one-year period in an average LTCF in Europe

The goal is to use the results of the study as parameters for the estimation of the burden of HAIs in LTCFs at national and European level, both in terms of estimating the number of infected residents per year as the number of disability-adjusted life years.

3 Methods

3.1 Design

One-year (12 months) prospective data collection, starting no later than 31st May 2022 and ending no later than 31st May 2023.

3.2 Survey population

3.2.1 Countries

All EU/EEA Member States are invited to participate through ECDC's surveillance of healthcare-associated infections network (HAI-Net).

3.2.2 National survey coordinators

In each country, one or more national survey coordinators (NSCs) are responsible for the recruitment of LTCFs, training activities for data collectors and the organisation of the longitudinal study in participating LTCFs (see section 5: role of the national survey coordinator).

3.2.3 Eligibility criteria for LTCFs

The term 'long-term care services' refers to the organisation and delivery of a broad range of services and assistance to people who are limited in their ability to function independently on a daily basis, i.e., to autonomously perform the basic activities of daily living, over an extended period of time. Additionally, there is often a need for basic medical services (wound dressing, pain management, medication, health monitoring, prevention, rehabilitation or palliative care). Long-term care comprises a mix of both health and social components, therefore pertaining to both health and social sectors.

LTCFs typically have residents who:

- need constant supervision (24 hours a day);
- need 'high-skilled nursing care', i.e., more than 'basic' nursing care and assistance for daily living activities;
- are medically stable and do not need constant 'specialised medical care' (i.e., care administered by specialised physicians);
- do not need invasive medical procedures (e.g., ventilation).

Inclusion of LTCFs: Preferably LTCFs with an existing continuous surveillance of infections or other register of infections should be included. Furthermore, LTCFs with a median length of stay which is approximately 12 months or more are preferred. In the HALT (2010) and HALT-2 (2013) projects, the most frequently reported LTCF types were general nursing homes, residential homes and mixed type facilities, respectively (Table 3). Therefore, LTCFs should be recruited to this survey in that order of preference.

Table 1 Description of LTCF types

LTCF types	Description
**General nursing homes**	In these facilities, residents need medical or skilled nursing and supervision 24 hours a day. These facilities principally provide care to older persons with severe illnesses or injuries.
**Residential homes**	In these facilities residents are unable to live independently. They require supervision and assistance for the activities of daily living (ADL). These facilities usually include personal care, housekeeping and three meals a day.
**Mixed LTCFs**	These facilities provide different types of care in the same facility.
**Other**	Other facilities, not classifiable among the above mentioned LTCF types.

Remark: This classification does not imply that the characteristics of residents within each facility type are strictly homogeneous.

The following facilities should be excluded: hospital long-term care wards, hostel care (hotel without any kind of nursing care), sheltered care houses, day centres, home-based centres, protected living.

3.2.4 Eligible residents

Inclusion of residents: All residents present on day 1 of the study period should be included. It is recommended to include residents that are planned to stay for at least the entire study period (e.g. short temporary stays planned for discharge during the follow-up period should not be included).

As many larger LTCFs have residents within distinct wards, buildings or departments, selection of a specific ward/department is also possible. In this case, all residents present on day 1 of the study period in the selected ward(s) should be included. For example, in this scenario, wards with the highest staff-to-resident ratio may be purposefully sampled, as residents in these wards are at highest risk of a HAI due to the higher prevalence of relevant risk factors.

New residents entering the facility or the selected ward(s) during the year should not be included. Residents discharged or deceased during the study should not be replaced by new residents.

3.2.5 Sample size

The targeted sample size for the longitudinal study is approximately 5 to 8 countries that collect data for and follow up 250 residents in one or more LTCFs per Member State for one year.

3.3 Data collection tools

Data are collected by using three questionnaires: an institutional questionnaire, a resident questionnaire and an infection questionnaire.

The **institutional questionnaire (annex 1)** collects identification data, structural characteristics, type of facility and study workload indicators (i.e. number of persons involved and working hours for the data collection).

The **resident questionnaire (annex 2)** should be used for data collection of the resident workload indicators, risk factors and COVID-19 history and vaccination at the start of the study and, during the follow-up period, to record any temporary discharge from the facility.

The **infection questionnaire (annex 3)** should be used for each infection that is developed by any eligible resident during the follow-up period.

- The **case definitions of infections (annex 4)** should be used to identify infections in eligible residents.
- The **code list for microorganisms (annex 5)** should be consulted when completing the infection questionnaire, to identify the appropriate codes for detected microorganisms and (optionally) their antimicrobial resistance profiles.

All data should be entered in an excel file provided by and sent to the NSC(s) for quality check, data extraction and upload on the study web-based repository.

3.3.1 Institutional questionnaire

The **institutional questionnaire (annex 1)** collects data related to each participating LTCF. Variables are described in table below.

Variable	Description
**Country**	The country where the participating facility is located.
**LTCFId**	LTCF identifier; it is a unique identifier/code for the LTCF allocated by the national survey coordinator
**Size (total beds)**	The total number of beds in the LTCF. Both occupied and non-occupied beds should be included. Beds shared by partners should be counted as two beds.
**Type of LTCF**	Type of LTCF according to the indications given by the national survey coordinator.
**Is medical resident care in the facility provided by?**	Who is responsible of the medical care in the facility according to following definitions: personal general practitioners (GP) or group practice(s) only; Medical staff, employed by the facility only; Both personal GPs/group practice(s) and medical doctor(s) employed by the facility.
**Are medical activities in the facility coordinated by a coordinating medical physician (CP)?**	In the facility there is a coordination of medical care in place according to following definitions: No, there is no internal or external coordination of the medical activity; Yes, there is a physician from inside the facility (internal) who coordinates the medical activities; Yes, there is a physician from outside the facility (external) who coordinates the medical activities; Yes, there is both a physician from inside and outside the facility (internal and external) who coordinates the medical activities
**Is a surveillance programme of healthcare-associated infections in place in the facility?**	The LTCF perform a surveillance of infections on a regular basis, for example (there is an annual summary report of the number of urinary tract infections, respiratory tract infections, etc…).
**Are laboratory tests routinely performed to diagnose an infection?**	In the LTCF, laboratory tests (e.g. microbiological exams) are routinely used to support the diagnosis of an infection. Note: the use of laboratory tests might depend on the infection type or on the physician’s attitude, please report the general practice for the most frequent infections.

Variable	Description
**Start date of the data collection**	The date of the start of the data collection.
**End date of the data collection**	The date of the end of the data collection.
**Function of the data collector(s)**	Report the profession of the data collector according to following definitions: Coordinating physician; Infection control doctor; Infection control nurse; Medical doctor/general practitioner; Head nurse; Registered nurse; Nursing assistant; Other.
**Hours for the data collection**	Report the total number of hours spent for the data collection by data collector.

3.3.2 Resident questionnaire

The resident questionnaire is used to collect data to describe the population, stratify the results, calculate risk indexes, adjust HAI mortality estimates. Data collectors should collect information from each resident who is registered as a resident in the facility on day one, according to eligibility criteria described in 3.2.4.

Important: name and surname of the residents are for internal use only, to facilitate internal tracking of data. These sensitive data must not be transferred to NSC(s) and/or EU study coordination centre (see 7. Ethical consideration and national data protection regulations).

Variable	Description
**Resident Name and Surname**	Name and surname of resident, for internal use, these must be deleted before data submission.
**ResidentID**	The pseudonymised unique study number for this resident. The link with the resident name must remain only at the LTCF level for data collection purposes only.
**Date of Admission**	The date of admission in the current LTCF. Enter the first date of admission to the LTCF, disregarding shorter periods of absence because of hospitalisation or other reasons, unless the resident was administratively considered discharged, and the room/bed of the resident was assigned to another person.
**Year of Birth**	Year the resident was born
**Gender**	Gender of the resident: Male or Female
**Disoriented**	Residents who suffer from periods of confusion especially as to time, place, or identification of persons (e.g., cognitive impairment) due to a chronic disease or condition diagnosed by a specialist or according to the mental evaluation scale used in the LTCF. Describe the condition according to following definitions: no (the resident is fully oriented); mild (1st tertile of the score in mental evaluation scale in use in the LTCF); moderate (2nd tertile of the score in mental evaluation scale in use in the LTCF) and severe (3rd tertile of the score in mental evaluation scale in use in the LTCF).
**Mobility**	Is the resident ambulant (i.e. he/she can walk alone with or without canes, crutches, walkers, etc), does he/she need a wheelchair for his/her movement or is he/she bedridden at the start of the follow-up period?
**Incontinence**	Presence of urinary and/or faecal incontinence. Lack of control of the sphincter from bladder or bowel resulting in an uncontrolled loss of urine or faeces and necessitating the use of diapers. A resident with a urinary catheter should not be considered as incontinent for urine.
**Urinary Catheter**	Presence of urinary catheter at the start of the follow-up period. Any tube system placed in the body to drain and collect urine from the bladder, e.g., an indwelling urinary catheter, suprapubic or abdominal wall catheter, a cystostomy. External catheters not draining urine directly from the bladder (e.g., condom catheters) should not be included.
**Vascular Catheter**	Presence of a vascular catheter at the start of the follow-up period. Any tube system placed in the body to access the vascular (venous, arterial) system, (e.g., a peripheral intravenous catheter, an implanted vascular access system or any other intravascular access system (including arteriovenous fistulae).
**Charlson's index**	Classification of the severity of underlying medical conditions, at the start of the follow-up period. Report any single condition if present. See next table for details.
**Date end follow up**	Date indicating the end of the follow-up for this resident; in principle end of study follow up period or earlier if resident deceased or was discharged before the end of the study.
**Status at end follow up**	The resident status (outcome) at the end of the follow-up period. Report if the resident is alive, resident is in LTCF; alive, but hospitalised at end of follow-up; alive, but discharged from the facility to e.g., home, family, not to hospital; alive, temporary discharge (not hospital); resident deceased or unknown status.
**Date of temporary discharge**	The date of discharge from the current LTCF (e.g. resident being hospitalised, resident returning home for a family visit or holiday). Only discharges for at least one night should be considered. Note: for all residents who have s/s in the first month of the study period, the temporary discharge form must be completed retrospectively (e.g. report the last temporary discharge prior to the start day).

Variable	Description
**Date of re-admission in the current LTCF**	The date the resident was readmitted in the current LTCF after a temporary discharge that lasted for at least one night.
**Place where discharged**	Home; hospital urgent; hospital planned; other LTCF
**Reason for the hospitalisation**	Describe the hospital stay specifying if the admission was for medical, surgical or diagnostic purposes.

Charlson's index variables

These variables must be collected at the beginning of the study for each resident. For each condition, a weight is assigned. In general, consider any official diagnosis made by a specialist. The score will be automatically calculated when the data are input in the electronic sheet.

Variable	Description	Assigned weight for disease
**Myocardial infarction**	Residents with one or more definite or probable myocardial infarction; these Residents had been hospitalised and had electrocardiographic and/or enzyme changes. Residents with electrocardiographic changes alone were not designated as having had an infarction.	1
Decompensatio cordis	Residents who have had exertional or nocturnal dyspnoea and who have responded symptomatically (or on physical examination) to digitals, diuretics, or afterload reducing agents. It does not include Residents who are on medication but have had no symptomatic response and no evidence of improvement of physical signs.	1
**Peripheral arterial disease**	Residents with intermittent claudication or those who had a bypass for arterial insufficiency, those with gangrene or acute arterial insufficiency, and those with untreated thoracic or abdominal aneurism (6 cm or more).	1
**Cerebrovascular disease**	Residents who have any history of or receive a treatment for a cerebrovascular disease including stroke, transient ischemic attack (TIA), aneurysm, and vascular malformation.	1
**Dementia**	Residents with chronic cognitive deficit officially diagnosed by a physician.	1
**Chronic lung disease**	Residents who are short of breath with slight activity, with or without treatment and those who are short of breath with moderate activity despite treatment. Also include Residents who are short of breath at rest, despite treatment, those who require constant oxygen, those with CO_2 retention.	1
**Peptic ulcer**	Residents who have any history of treatment for ulcer disease or history of ulcer bleeding.	1
**Liver disorder**		
Mild	Residents with chronic hepatitis or cirrhosis without portal hypertension.	1
Moderate/Severe	Cirrhosis and portal hypertension with variceal bleeding history.	3
**Diabetes**		
Mild	Residents who had previous hospitalisations for ketoacidosis, hyperosmolar coma, or control and those with juvenile onset or brittle diabetics. Also include all other diabetes treated with insulin or oral hypoglycaemic but not diet alone.	1
Moderate/Severe	Residents with retinopathy, neuropathy, or nephropathy.	2
**Hemiplegia**	Residents with the hemiplegia or paraplegia, whenever it occurred as a result of a cerebrovascular accident or other condition.	2
**Kidney disorders**	Residents with serum creatinine > 2 mg% or Residents on dialysis, those who had a transplant, and those with uraemia.	2
**Chronic urinary disorders**	Patient with any chronic conditions that affects the urinary tract and is not caused by an acute infection (e.g., polyuria, nocturia, etc…).	1
**Malignancies**	Residents with solid tumours without documented metastases, but initially treated in the last five years, including breast, colon, lung and a variety of other tumours.	2
**Leukaemia**	Residents with acute and chronic myelogenous leukaemia, acute and chronic lymphocytic leukaemia, and polycythaemia vera.	2
**Lymphoma**	Residents with Hodgkin's lymphoma, Waldestrom's macroglobulinemia, myeloma, and other lymphomas.	2
**Metastasis**	Residents with metastatic solid tumours, including breast, lung, colon and other tumours.	6
**Systemic disease**	Residents with systemic lupus erythematous, polymyositis, mixed connective tissue disease, polymyalgia rheumatic, and moderate to severe rheumatoid arthritis.	6

COVID-19 variables

These variables must be collected at the beginning of the study for each resident. An update can be required according to national vaccination plans.

Variable	Description
History of infection	
**Previous COVID-19 episode**	Report if the resident had a laboratory confirmed COVID-19 episode in the months prior the start of the study.
**Date of the previous COVID-19 episode**	Report month and year of the start of the last known COVID-19 episode in the resident.
**Disease severity of previous COVID-19 episode**	Report the stage of the disease (worst moment): asymptomatic, symptomatic: mild to moderate (signs and/or symptoms compatible with COVID-19 without need for oxygen therapy and oxygen saturation ≥ 92% or unknown), symptomatic: severe (i.e., the resident needed oxygen therapy for shortness of breath due to COVID-19 and/or oxygen saturation < 92%), unknown.
**More than one episode**	Report if the resident had more than one laboratory confirmed COVID-19 episode, prior to the last one reported.
Vaccination	
**COVID-19 vaccine offered**	Report if the vaccine for COVID-19 was offered to the resident.
**Contraindication for the COVID-19 vaccine**	If the resident did not receive a vaccine, report if there were any contraindications (e.g., due to documented allergy to the vaccine or one of its compounds) or if the resident (or his/her caregiver) refused the vaccine.
**First dose of COVID-19 vaccine**	Report if the resident received a first dose of a vaccine for COVID-19.
**Date of the first dose**	Report the date of the dose administration.
**Brand name of first dose vaccine received (product name)**	Report the brand name (product name) of the first of dose vaccine received.
**Mode of vaccine ascertainment (how the interviewer verified the vaccination status)**	Mode of vaccine ascertainment (how the interviewer verified the vaccination status: not documented; self-report; vaccination card; vaccination registry; other).
**Second dose of COVID-19 vaccine**	Report if the resident received a second dose of a vaccine for COVID-19 (if applicable). If not, report the reason (e.g., if the vaccine administered in the first dose was a single dose or if the resident had a previous COVID-19 episode within 6 months from the first dose administration or if the resident, for any other reason, did not receive the second dose even if it was required according to that vaccine plan).
**Date of the second dose**	Report the date of the dose administration (if applicable).
**Brand name of the second dose vaccine received (product name)**	Report the brand name (product name) of the second dose of vaccine received.
**Mode of vaccine ascertainment of the second dose (how the interviewer verified the vaccination status)**	Mode of vaccine ascertainment (how the interviewer verified the vaccination status: not documented; self-report; vaccination card; vaccination registry; other)
**Third dose of COVID-19 vaccine**	Report if the resident received a third dose of a vaccine for COVID-19 (if applicable). If not, report the reason (e.g., if the vaccine administered in the first dose was a single dose or if the resident had a previous COVID-19 episode within 6 months from the first dose administration or if the resident, for any other reason, did not receive the second dose even if it was required according to that vaccine plan).
**Date of the third dose**	Report the date of the dose administration (if applicable).
**Brand name of the third dose vaccine received (product name)**	Report the brand name (product name) of the second dose of vaccine received.
**Mode of vaccine ascertainment of the third dose (how the interviewer verified the vaccination status)**	Mode of vaccine ascertainment (how the interviewer verified the vaccination status: not documented; self-report; vaccination card; vaccination registry; other)

3.3.3 Infection questionnaire

This form is used to collect case-based Infection data. Record the following information for each infection identified using the decision algorithm **(annex 3)**:

Variable	Description
**Resident Name and Surname**	Name and surname of resident, for internal use, these must be deleted before data submission.
**ResidentID**	The pseudonymised unique study number for this resident. The link with the resident name must remain only at the LTCF level for data collection purposes only.
**Infection Site**	Code of the infection site. See annex for descriptions and definitions.
**If 'OTHER', please specify**	If infection code='OTHER', please provide more information on the type of infection.
**Infection diagnosed in hospital**	Report if the infection was diagnosed or confirmed during a hospitalisation.
**Date of onset**	Date of onset of the infection (dd/mm/yyyy). Not to be recorded if signs/symptoms are present at (re-)admission but should be completed if onset during current stay in the LTCF. Record the date (order of priority): 1) first signs or symptoms of the infection, or 2) the date of the diagnosis by the attending physician, or 3) the starting date of the treatment, or 4) the date the first sample tested positive was taken, or 5) estimate the date if none of the previous is available.
**End date**	End date of the infection (order of priority): 1) End of the treatment, or 2) declaration of recovery by the attending physician, or 3) complete remission of clinical signs and symptoms, or 4) the date the first negative sample was taken, or 5) estimate the date if none of the previous is available.
**Infection outcome**	The status of the resident at the end of the infection episode. Report if the resident is alive, death or it was not possible to assess, according to follow definitions: Alive, the resident is alive at the end of the follow-up period; Sole cause - infection was the sole cause of death – no other disease or condition causing the death was present (sufficient condition); part of the causal sequence - infection was part of the causal sequence of events that led to death but not sufficient on its own; contributory cause - infection was a contributory cause but not related to the disease or condition causing the death; No contribution - infection did not contribute to the death or the contribution was redundant, i.e. the patient would have died anyway; Unknown or not verified - Contribution of HAI to death of the patient unknown or not verified; Status of the resident is unknown or not verified.
**Microorganism**	See paragraph 3.3.3.2 and annex 5 for description and codes
**Antimicrobial resistance**	See annex 3.5
**Comments**	Report any relevant comment which might further describe the infection episode (e.g., it is a known recurrent infection).

3.3.3.1 Infections

Data collectors must identify eligible residents presenting signs and/or symptoms of an infection[a] or with a positive laboratory test for COVID-19[b]. During data analysis, the infections will be classified as LTCF acquired (current or other LTCF), hospital or community acquired according to the timing of the temporary discharge. Please, carefully record every temporary discharge (as the date of temporary discharge and date of readmission) for each resident included in the study, even if he/she is not experiencing and infection.

Notes:

[a] Chronic symptoms, such as cough or urinary urgency, are commonly not associated with infection. Non-infectious causes should always be considered before a diagnosis of infection is made. A change in the resident's status (s/s new or acutely worsening) is an important indication that an infection is in development.

[b] Covid-19 diagnosis is made on the sole confirmation of a documented laboratory test (viral RNA target or antigenic detection from an oropharyngeal or nasal swab or any other appropriate clinical specimen) even in the absence of any clinical signs and symptoms.

3.3.3.2 Identifying the infection code using the decision algorithms

By comparing eligible resident's signs/symptoms with those listed in the decision algorithms (annex 4), data collectors will see whether enough signs/symptoms are present to confirm an infection and will enter the relevant code(s) onto the infection questionnaire within 'infection code'. Therefore, exhaustive searching for signs/symptoms present in residents is crucial.

The decision algorithms used in this survey are based on clinical criteria, i.e., CDC/SHEA case definitions[1] which in turn are based on the McGeer[2] criteria for the surveillance of infections in LTCFs.

Definitions of key terms used in the decision algorithms

Key terms	Description/definition
Fever	1.Single >37.8°C oral/tympanic membrane* OR 2. Repeated >37.2°C oral or >37.5°C rectal OR 3. >1.1°C over baseline from any site (oral, tympanic, axillary)<br>* tympanic membrane = membrane that separates the external ear from the middle ear.
Leucocytosis	1. Neutrophilia > 14 000 leucocytes/mm^3 OR 2. left shift (>6% bands or ≥ 1500 bands/mm^3) {Stone, 2012 #113}
Acute change in mental status	Acute onset + fluctuating course + inattention AND either disorganised thinking or altered level of consciousness
Acute functional decline	New three-point increase in total ADL score (Range 0–28) from baseline based on seven ADL items (bed mobility, transfer, locomotion, dressing, toilet use, personal hygiene, eating) each scored from 0 (independent) to 4 (total dependence) OR increased dependency defined by scales other than ADL
**Urinary tract infection**	Can be an infection of the kidney, ureter, bladder or urethra
Costovertebral angle pain	Pain in the area of the back overlying the kidney (between the 12th rib and the spine)
Suprapubic pain/tenderness	Pain or tenderness in the area above the pubis
**Respiratory tract infection**	Can be an infection of the upper or lower respiratory tract
Upper respiratory tract infection	Infection of the (naso-)pharynx ((naso-)pharyngitis) or tonsils (tonsillitis)
Lower respiratory tract infection	Infection of the trachea and bronchus (bronchitis), bronchiole (bronchiolitis) or lung and alveoli (pneumonia)
Lymphadenopathy	Disease of the lymph nodes (swollen or enlarged)
Infiltrate	Deposition of fluid (e.g. blood, pus, etc.) in tissues and cells
Sputum	Secretion expectorated from the lower respiratory tract (not to be confused with saliva)
Pleuritic chest pain	Pain in the chest during inhalation which can cause fast and superficial breathing to decrease the pain
**Skin infections**	Infections of the skin
Cellulitis	Infection of the connective tissue
Soft tissues	Tissues that connect support or surround other structures or organs (muscles, tendons, ligaments, nerves, blood vessels, fat, fibrous tissues, fascia and membranes)
Maculopapular rash	Rash characterised by spots and bumps
Herpes simplex	Disease caused by a virus leading to a rash (often around the lips and nose) with groups of blisters containing fluid which soon dry out
Herpes zoster	Disease caused by a virus; mostly painful blister-shaped rash in areas where many sensory nerves are present (e.g. face, chest, shoulders and hip)
Scabies	Contagious and heavy itching disease of the skin caused by a mite
**Gastrointestinal infection**	Infection of the stomach and/or intestines
Clostridioides (Clostridium) difficile (CD)	*C. difficile* (gram-positive sporulating bacilli); can cause persistent diarrhoea and ulcero-haemorrhagic colitis
Toxic megacolon	Life-threatening complication that causes widening (dilation) of the large intestine and symptoms such as abdominal pain, distension, tenderness, fever, rapid heart rate and can even lead to shock
Pseudomembranous colitis	A cause of antibiotic-associated diarrhoea (often caused by *C. difficile*) characterised by abdominal cramps, bloody stools, fever and diarrhoea
**Eye infection**	Infections of the eye
Conjunctival erythema	Redness of the conjunctiva (mucous membrane lining the eyelid)

[1] Stone ND, Ashraf MS, Calder J, Crnich CJ, Crossley K, Drinka PJ, et al; for the Society for Healthcare Epidemiology Long-Term Care Special Interest Group. Surveillance definitions of infections in long-term care facilities: Revisiting the McGeer criteria. Infect Control Hosp Epidemiol. 2012;10:965-977.

[2] McGeer A, Campbell B, Emori TG, Hierholzer WJ, Jackson MM, Nicolle LE, et al.. Definitions of infection for surveillance in long-term care facilities. Am J Infect Control. 1991;19:1-7.

Infection codes

Infection	Level	Infection code
**Urinary tract infections (UTIs)**	Confirmed / Probable	UTI-C / UTI-P
**Respiratory tract infections (RTIs)**		
Common cold syndromes/pharyngitis	Confirmed	COLD
Influenza-like illness ('Flu')	Confirmed	FLU
Pneumonia	Confirmed	PNEU
Other lower RTI	Confirmed	LRTI
**COVID-19**		
Asymptomatic	Confirmed	COV-ASY
Mild/moderate	Confirmed	COV-MM
Severe	Confirmed	COV-SVR
**Surgical site infections (SSIs)**		
Superficial incisional SSI	Confirmed	SSI-S
Deep incisional SSI	Confirmed	SSI-D
Organ/space SSI	Confirmed	SSI-O
**Skin infections**		
Cellulitis/soft tissue/wound infection	Confirmed	SKIN
Scabies	Confirmed	SCAB
Herpes simplex or herpes zoster infection	Confirmed	HERP
Fungal infection	Confirmed	FUNG
**Gastrointestinal tract infections**		
Gastroenteritis	Confirmed	GE
Clostridium difficile infection	Confirmed	CDI
**Eye, ear, nose and mouth infections**		
Conjunctivitis	Confirmed	CONJ
Ear infection	Confirmed	EAR
Sinusitis	Confirmed	SINU
Mouth infection or oral candidiasis	Confirmed	ORAL
**Bloodstream infections**	Confirmed	BSI
**Unexplained febrile episode**	Confirmed	FUO
**Other infection(s)**		OTHER

Notes for COVID-19 infection reporting

In this study, COVID-19 case definition is based on the positive result of a laboratory test from an appropriate clinical specimen. The infection should be reported even for asymptomatic cases and therefore it is an exception to the indications given for the other infections.

In the case in which the resident is tested positive for COVID-19 and symptomatic it is asked to record, in addition, the clinical manifestation according to other study case definitions. For the second infection, it is recommended to report the associated microorganism as VIRCOV.

As example:
The resident has a documented laboratory confirmation test for COVID-19 (viral RNA target or antigenic detection from an oropharyngeal or nasal swab or any other appropriate clinical specimen).
AND
The resident develops signs and symptoms compatible with a Pneumonia, confirmed according to case definition.

The case should be reported in two distinct infection questionnaires, as follows:

(a) One questionnaire for COVID-19, recording the infection as COV-MM or as COV-SVR (depending on the need of oxygen therapy and/or blood oxygen saturation level, see definition);

(b) One questionnaire for the Pneumonia, recording as PNEU, indicating VIRCOV in the field microorganism, in addition to other isolated microorganisms, if any.

3.3.3.3 Isolated microorganism and antimicrobial resistance

Data on the isolated microorganism(s) and (optionally) antimicrobial resistance are collected in the infection questionnaire. It is recognised that there is a low frequency of laboratory testing of clinical samples from LTCFs in Europe, and differences between the antimicrobial susceptibility testing protocols used by those laboratories.

Collect microbiological results available by the end of the infection event follow-up. Specify up to three isolated microorganisms, using the microorganism code list (annex 5). If no microbiological result is available, one of the following options should be selected:

**_NOEXA**	EXAMINATION NOT DONE	No diagnostic sample taken; no microbiological examination done
**_NA**	RESULTS NOT AVAILABLE	The results of the microbiological examination are not available or cannot be found
**_NONID**	MICROORGANISM NOT IDENTIFIED	Evidence exists that a microbiological examination has been done, but the microorganism cannot be correctly classified
**_STERI**	STERILE EXAMINATION	A microbiological examination has been done, but the result was negative (e.g., negative culture)

OPTIONAL: Five groups of selected bacteria (highlighted in red in the microorganism code list) can have their antimicrobial resistance reported according to their resistance profile as indicated in the table below.

Antimicrobial resistance codes and profiles

Microorganism	Tested antibiotic	Antimicrobial resistance			
Staphylococcus aureus (STAAUR)	Oxacillin (OXA)	Susceptible (S)	–	Resistant (R)	Unknown (U)
	Glycopeptides (GLY)	Susceptible (S)	Intermediate (I)	Resistant (R)	Unknown (U)
Enterococcus* species** (ENC)	Glycopeptides (GLY)	Susceptible (S)	Intermediate (I)	Resistant (R)	Unknown (U)
**Enterobacteriaceae**[1], including:<br>*Escherichia coli (ESCCOL)*<br>*Klebsiella* species (KLE***)<br>*Enterobacter* species (ENB***)<br>*Proteus* species (PRT***)<br>*Citrobacter* species (CIT***)<br>*Serratia* species (SER***)<br>*Morganella* species (MOGSPP)	Third-generation cephalosporins (C3G)	Susceptible (S)	Intermediate (I)	Resistant (R)	Unknown (U)
	Carbapenems (CAR)	Susceptible (S)	Intermediate (I)	Resistant (R)	Unknown (U)
Pseudomonas aeruginosa (PSEAER)	Carbapenems (CAR)	Susceptible (S)	Intermediate (I)	Resistant (R)	Unknown (U)
Acinetobacter baumannii (ACIBAU)	Carbapenems (CAR)	Susceptible (S)	Intermediate (I)	Resistant (R)	Unknown (U)

[1] Antimicrobial resistance markers are <u>not</u> collected for other Enterobacteriaceae (e.g., Hafnia spp., Salmonella spp., Shigella spp., Yersinia spp.); OXA: susceptibility to oxacillin, or other marker of meticillin-resistant S. aureus (MRSA), such as cefoxitin, cloxacillin, dicloxacillin, flucloxacillin, meticillin; GLY: susceptibility to glycopeptides: vancomycin or teicoplanin; C3G: susceptibility to third-generation cephalosporins: cefotaxime, ceftriaxone, ceftazidime; CAR: susceptibility to carbapenems: imipenem, meropenem, doripenem.

3.3.4 Flowchart summarising the data collection process

In the days prior to the beginning of the study

1. Complete the institutional questionnaire

1. Complete the institutional questionnaire (i.e., main protocol Annex 1)
2. Insert data into the excel spreadsheet file (tab.1 – institutional form).

2. Prepare the resident questionnaire

1. Complete the columns name and surname in the resident questionnaire.
2. Prepare all relevant data sources regarding the resident's health status and record all the required information for each resident.
3. Once the data collection is completed for all residents, insert data into the electronic spreadsheet provided by the National Study Coordinator (delete the name and surname before sending).
4. Check the completeness of data, save the file and send it to the National Study Coordinators and/or to the EU Study Coordinator.

During the follow-up period

1. Check the eligible residents for signs/symptoms of infections and temporary discharges

1. Check for any systemic signs and/or symptoms of infections (e.g., new onset fever).
2. Check for any local signs and/or symptoms (e.g., gross haematuria, new or marked increase in frequency of urination).
3. Check for any pharmacological treatment for an infection.
4. Check for any diagnostic test result suggestive for an infection (e.g. positive urine culture, positive urinary dipstick, COVID-19).
5. Check for any temporary discharge or re-admission in the LTCF.

2. Diagnose infections according to the case definitions of infections

1. Apply case definitions of infections according to study protocol (see annex).
2. Open a new infection form, apply resident's identifiers (name, surname and ResidentID)
3. Record the site of infection (see infection codes) and start date of signs/symptoms.
4. Complete the form recording end date of infection when the infection is resolved (complete remission of signs and symptoms, end of pharmacological treatment or death).
5. Record any relevant information on the microbiological culture available at the end of the follow up of the infection.
6. If the infection is diagnosed, record information into the excel infection form (see annex).
7. Delete name and surname in the excel infection form and send it to the National Study Coordinator for upload on the study's Sharepoint.

3. Input data into data collection tools

1. At the end of data collection, input all collected information into the appropriate sheet in the excel file.
2. Verify completeness of data.
3. Check and remove any sensitive data (name and surname of residents).

End of data collection

1. **Finalise data collection.**
2. **Review missing data according to reports from NSC(s).**

The data collection tools include an electronic spreadsheet in Microsoft Excel. This file consists of 6 sheets, one for each set of information to collect, in particular:

1. Institutional questionnaire – which records information about the participating LTCF, relevant for the data collection.
2. Resident list – records individual risk factors for each eligible resident. This information must be recorded at the beginning of the study. Resident's name and surname are for internal use only (to facilitate recognition by the LTCF staff) and must be deleted before the file is sent to the NSC(s).
3. Charlson's index variables – this is a separate part of the resident list (automatically generated) and used to record and calculate the Charlson's index for each resident. The index is calculated automatically and reported in the corresponding column in the resident list. This information must be recorded at the beginning of the study.
4. Covid-19 – this is the list in which all relevant information about history of covid-19 and vaccination can be recorded. This information must be recorded at the beginning of the study.
5. Temporary discharges – this form should be used for recording of any temporary discharge (for at least one night) of a resident from the LTCF. Resident ID must be selected from the menu in the corresponding column (name and surname will be automatically generated). This information must be recorded at the beginning of each temporary discharge and completed when the resident returns to the LTCF.
6. Infection questionnaire – this form is used to facilitate the recording of variables for each episode of an infection in the eligible resident population. Once all information are included in the form, a string is automatically generated (already in the required format) that can be copied & pasted into the infection list.
7. Infection list – this is the list used to record all infection occurring in the eligible residents. Resident IDs are automatically generated. The infection string must be pasted to the same row, next to the previous one.

The excel file is editable only in allowed cells, all other fields cannot be modified. To ensure standardisation of data collection, variables can be selected within a list in a dropdown menu or automatically calculated. Other variables will be manually edited.

3.4 Data collectors

Depending on the available resources, data can be collected by local data collectors (e.g., designated physician, infection control doctor/nurse, head nurse, etc.) or local data collectors supported by an external data collector (i.e., person recruited by the NSC(s), e.g., doctor, infection control nurse or the NSC him/herself).

Both local and external trained data collectors should visit the facility periodically to review each resident with the nurse in charge, nurses' aide and healthcare workers of the LTCF, looking for recent symptoms suggestive of infection, examining charts, case notes and drug charts. Residents with suspected infection(s) and residents receiving antimicrobial agents should be further reviewed and discussed with the attending physician if possible.

Training material is developed by the HALT-4 coordination group. Training of NSCs and local data collectors is strongly recommended.

4. Training

Online training workshop(s) will be held prior to the start of the survey period and should be attended by nominated persons from the participating EU/EEA Member States. Training material for the local/external data collectors is available from ECDC (in English). It is recommended that national/regional survey coordinators organise at least one one-day information and training session for LTCFs participating in the study prior to the national/regional survey.

5. Role of the national survey coordinator

National survey coordinators (NSCs) are crucial determinants of the success of the study.

Their tasks before data collection include the following:

- select and invite LTCFs to participate;
- participate in ECDC's training workshop;
- organise at least a one-day information and training session for LTCFs participating in study;
- distribute the data collection tools (e.g., data collection spreadsheet tools);
- (if required) translate data collection tools and letters into national languages.

Their tasks during the study period include the following:

- assist LTCFs during data collection (helpdesk);
- collect and transfer the local databases to the EU study coordination centre;
- monitor local data collection and verify periodically the accuracy and reliability of the data collection.

Their tasks after data collection include the following:

- share the feedback to the participating LTCF;
- review scientific reports or research articles based on the longitudinal study.

6. Data delivery

6.1 Electronic data collection tools

Electronic data collection tools are based on Microsoft Excel spreadsheet files and are intended for use at local (LTCF) level and allow data transfer to the NSC and EU study coordinator.

All data are stored on the local computer rather than in a central database. Therefore, data need to be prepared for sending by deleting name and surname from each file in which this information is collected (resident list and infection questionnaire). A copy of the saved file can be sent to the NSC following instruction given by the EU study coordination centre.

Once all data are checked for completeness, NSCs should send every file according to instructions provided by the EU study coordinator.

6.2 Deadline for data delivery

The LTCF databases should be sent to the NSCs and then shared with ECDC using an agreed, secure method (e.g. an sftp server of ECDC), every two weeks in the first 3 months of the study. Thereafter, the timing of the data delivery will be planned according to needs of the country and LTCF.

6.3 Data analysis and feedback

All single LTCF databases will be aggregated into a European database, containing data from all national databases and will be checked for errors and inconsistencies by ECDC and/or the EU study coordination centre. Individual feedback reports (in English) will be generated for each participating LTCF and sent to the NSC for further distribution (e.g. to each participating LTCF).

A national report will be prepared using aggregated results, sent to NSCs from each participating country for verification.

Final results of the study will be published, in the form of a paper, on an indexed journal.

6.4 Data ownership

NSCs are encouraged to publish their data in international peer-reviewed journals and/or present their results at international conferences. The work done by the NSCs and the LTCFs should be acknowledged, e.g. by adding 'on behalf of the national networks' to the author list and/or by thanking all NSCs by name in the acknowledgements section. ECDC should be acknowledged in all scientific publications (including posters and oral communications).

All analyses and outputs, including data other than their own country's data, should be performed in consultation and in agreement with ECDC. All scientific outputs should be communicated to ECDC in advance of publication; these may be referred to on the ECDC website, and/or in other public outputs.

7. Ethical considerations

Member States will have different requirements for ethical approval for a longitudinal surveillance in LTCFs. The experience from the HALT (2010), HALT-2 (2013) and HALT-3 (2016-2017) projects is that some countries required approval from an ethics committee. Some of the committees requested that written consent be obtained from each resident with an infection, or if not possible (e.g., in case of cognitive impairment) from a 'proxy' such as a carer or a medical professional. Data collectors in these Member States found that it was relatively feasible to acquire the signatures, as simply explaining the necessity of the study to the resident or their 'proxy' was sufficient.

Confidentiality of LTCF data and resident data is assured by:

NSCs attributing an LTCF survey number to each participating LTCF. The participating LTCFs will not be identifiable by other LTCFs/persons since all reports and presentations will only use LTCF survey numbers and never LTCF names. The key to the LTCF names from the LTCF survey number will not be sent to ECDC.

A unique, pseudonymised resident survey number will be allocated to each resident for whom a questionnaire is completed.

The resident list and the infection questionnaire include resident identifiers (name and surname) that must be removed before sharing databases with NSCs and/or EU coordination centre. The local files must be kept in the LTCF in a secure and confidential manner and should be destroyed at the end of the project, i.e., December 2023.

Data collected within the framework of the project should not be used for purposes other than those described in the objectives of the present protocol.

8. References

1. European Centre for Disease Prevention and Control. Point prevalence survey of healthcare-associated infections and antimicrobial use in European long-term care facilities. May–September 2010. Stockholm: ECDC; 2014. Available from https://ecdc.europa.eu/sites/portal/files/media/en/publications/Publications/healthcare-associated-infections-antimicrobial-consumption-point-prevalence-survey-long-term-care-facilities-2010.pdf

2. European Centre for Disease Prevention and Control. Point prevalence survey of healthcare-associated infections and antimicrobial use in European long-term care facilities. April–May 2013. Stockholm: ECDC; 2014. Available from http://ecdc.europa.eu/sites/portal/files/media/en/publications/Publications/healthcare-associated-infections-point-prevalence-survey-long-term-care-facilities-2013.pdf
3. Suetens C. Healthcare-associated infections in European long-term care facilities: how big is the challenge? Euro Surveill. 2012 Aug 30;17(35)
4. Broex E, Jans B, Latour K, Goossens H, ESAC Management Team. European Surveillance of Antimicrobial Consumption (ESAC): Results from the national survey of characteristics of nursing homes. Brussels: The Scientific Institute of Public Health (WIV-ISP); October 2010. ISBN: 9789057283017. Available from: http://www.nsih.be/BCKUP201509/download/nursing_homes/ESAC_NATSURV_NH.pdf
5. European Centre for Disease Prevention and Control. Protocol for point prevalence surveys of healthcare-associated infections and antimicrobial use in European long-term care facilities – version 2.1. Stockholm: ECDC; 2016. Available from https://ecdc.europa.eu/sites/portal/files/media/en/publications/Publications/HALT-3-LTCF-PPS-Protocol-v2.1.pdf
6. Cassini A, Plachouras D, Eckmanns T, Abu Sin M, Blank HP, Ducomble T, Haller S, Harder T, Klingeberg A, Sixtensson M, Velasco E, Weiß B, Kramarz P, Monnet DL, Kretzschmar ME, Suetens C. Burden of Six Healthcare-Associated Infections on European Population Health: Estimating Incidence-Based Disability-Adjusted Life Years through a Population Prevalence-Based Modelling Study. PLoS Med. 2016 Oct 18;13(10):e1002150.

Annex 1. Institutional questionnaire

Surveillance of healthcare-associated infections and antimicrobial use in European long-term care facilities (HALT)

One-year incidence of infections in long-term care residents: a prospective study

Institution Questionnaire

LTCFs questionnaire

Country		
LTCF Id		*Unique LTCF identification code given by the National coordinator*

LTCF characteristics

**Size (total beds)**			*Total number of beds in the facility at the beginning of the study*
**Type of LTCF**	q General Nursing home<br>q Mixed type facility	q Residential home<br>q Other	*Total number of beds in the facility at the beginning of the study*

**Are medical activities in the facility coordinated by a coordinating medical physician (CP)?**	❑ No, there is no internal or external coordination of the medical activity<br>❑ Yes, there is a physician from inside the facility (internal) who coordinates the medical activities<br>❑ Yes, there is a physician from outside the facility (external) who coordinates the medical activities<br>❑ Yes, there is both a physician from inside and outside the facility (internal and external) who coordinates the medical activities	*The Coordinating physician (CP) is a medical doctor in charge of the coordination of medical activities and standardisation of practices/policies in the facility.*
**Is a surveillance programme of healthcare-associated infections in place in the facility?**	❑ No<br>❑ Yes	*The LTCF perform a surveillance of infections on a regular basis, for example (there is an annual summary report of the number of urinary tract infections, respiratory tract infections, etc…).*
**Are laboratory tests routinely performed to diagnose an infection?**	❑ No<br>❑ Yes	*In the LTCF, laboratory test (e.g. microbiological exams) are routinely used to support the diagnosis of an infection. Note: the use of laboratory tests might depend on the infection type or on the physician attitude, please report the general practice for the most frequent infections.*

Survey dates

**From:**		*The date of the first day of data collection*
**To:**		*The date of the last day of data collection*

Study feasibility & workload

At the end of the survey, please report

**Total hours spent for the survey**	

Optional, please, specify the function, within the facility, of each data collector and the total hours spent for data collection

	Function	***Total hours for data collection***
**Data collector 1**	❑ Coordinating physician ❑ Infection control doctor ❑ Infection control nurse<br>❑ Medical doctor/general practitioner ❑ Head nurse ❑ Registered nurse	

	❑ Nursing assistant ❑ Other	
**Data collector 2**	❑ Coordinating physician ❑ Infection control doctor ❑ Infection control nurse<br>❑ Medical doctor/general practitioner ❑ Head nurse ❑ Registered nurse<br>❑ Nursing assistant ❑ Other	
**Data collector 3**	❑ Coordinating physician ❑ Infection control doctor ❑ Infection control nurse<br>❑ Medical doctor/general practitioner ❑ Head nurse ❑ Registered nurse<br>❑ Nursing assistant ❑ Other	
**Data collector 4**	❑ Coordinating physician ❑ Infection control doctor ❑ Infection control nurse<br>❑ Medical doctor/general practitioner ❑ Head nurse ❑ Registered nurse<br>❑ Nursing assistant ❑ Other	
**Data collector 5**	❑ Coordinating physician ❑ Infection control doctor ❑ Infection control nurse<br>❑ Medical doctor/general practitioner ❑ Head nurse ❑ Registered nurse<br>❑ Nursing assistant ❑ Other	

Comments

**Please, report any comment**

Max 800 characters

Annex 2. Resident questionnaire

Surveillance of healthcare-associated infections and antimicrobial use in European long-term care facilities (HALT)

One-year incidence of infections in long-term care residents: a prospective study

Resident Questionnaire

Resident identifiers

Resident Name			
Resident Surname			
ResidentID			
Gender	❑ Male	❑ Female	❑ Unknown
Year of Birth	*yyyy*		
Date of admission in the LTCF	*dd/mm/yyyy*	________/_______/_______	

Resident's risk factors

**Disoriented**	❑ No	❑ Mild	❑ Moderate	❑ Severe	❑ Unknown
**Mobility**	❑ Ambulant	❑ Wheelchair	❑ Bedridden	❑ Unknown	
**Incontinence**	❑ Yes ❑ No	❑ Unknown			
**Urinary Catheter**	❑ Yes ❑ No	❑ Unknown	**Vascular Catheter**	❑ Yes ❑ No	❑ Unknown

Comorbidities

**Myocardial infarction**	❑ Yes	❑ No	***Decompensatio cordis***	❑ Yes	❑ No
**Peripheral arterial disease**	❑ Yes	❑ No	**Cerebrovascular disease**	❑ Yes	❑ No
**Dementia**	❑ Yes	❑ No	**Chronic lung disease**	❑ Yes	❑ No
**Peptic ulcer**	❑ Yes	❑ No	**Liver disorder**	❑No ❑ Mild ❑ Moderate/Severe	
**Diabetes**	❑No ❑ Mild ❑ Moderate/Severe		**Hemiplegia**	❑ Yes	❑ No
**Kidney disorders**	❑ Yes	❑ No	**Chronic urinary disorders**	❑ Yes	❑ No
**Malignancies**	❑ Yes	❑ No	**Leukaemia**	❑ Yes	❑ No
**Lymphoma**	❑ Yes	❑ No	**Metastasis**	❑ Yes	❑ No
**Systemic disease**	❑ Yes	❑ No			

COVID-19

History of infection				
**Previous COVID-19 episode**	❑ Yes	❑ No	❑ Unknown	
**Date of the previous COVID-19 episode**	*dd/mm/yyyy*	_______ /_______/_____________		
**Disease severity of previous COVID-19 episode**	❑ asymptomatic	❑ Mild	❑ Moderate	❑ Severe
**More than one episode**	❑ Yes	❑ No	❑ Unknown	
Vaccination				
Pre-follow-up *(these is the status of the resident at the beginning of the follow-up)*				
**COVID-19 vaccine offered**	❑ Yes ❑ No ❑ Unknown			

**Contraindication for the COVID-19 vaccine**	❑ Yes ❑ No ❑ Unknown		

**First dose of COVID-19 vaccine**	❑ Yes ❑ No ❑ Unknown	**Brand name of first dose vaccine received**	____________________
**Date of the first dose**	________/________/___________	**Mode of vaccine ascertainment**	❑ not documented ❑ self-report ❑ vaccination card ❑ vaccination registry ❑ other

**Second dose of COVID-19 vaccine**	❑ Yes ❑ No ❑ Unknown	**Brand name of the second dose vaccine**	
**Date of the second dose**		**Mode of vaccine ascertainment of the second dose**	❑ not documented ❑ self-report ❑ vaccination card ❑ vaccination registry ❑ other

**Third dose of COVID-19 vaccine**	❑ Yes ❑ No ❑ Unknown	**Brand name of the third dose vaccine**	
**Date of the third dose**		**Mode of vaccine ascertainment of the third dose**	❑ not documented ❑ self-report ❑ vaccination card ❑ vaccination registry ❑ other

Post-follow-up *(this is the status of the resident at the end of the follow-up)*

**COVID-19 vaccine offered**	❑ Yes ❑ No ❑ Unknown	
**Contraindication for the COVID-19 vaccine**	❑ Yes ❑ No ❑ Unknown	

**First dose of COVID-19 vaccine**	❑ Yes ❑ No ❑ Unknown	**Brand name of first dose vaccine received**	____________________
**Date of the first dose**	________/________/___________	**Mode of vaccine ascertainment**	❑ not documented ❑ self-report ❑ vaccination card ❑ vaccination registry ❑ other

**Second dose of COVID-19 vaccine**	❑ Yes ❑ No ❑ Unknown	**Brand name of the second dose vaccine**	
**Date of the second dose**		**Mode of vaccine ascertainment of the second dose**	❑ not documented ❑ self-report ❑ vaccination card ❑ vaccination registry ❑ other

**Third dose of COVID-19 vaccine**	❑ Yes ❑ No ❑ Unknown	**Brand name of the third dose vaccine**	
**Date of the third dose**		**Mode of vaccine ascertainment of the third dose**	❑ not documented ❑ self-report ❑ vaccination card ❑ vaccination registry ❑ other

End of the Follow-up

**Date of the end of the follow-up**	____________________/ ____________________/ ____________________	
**Status at the end of the follow up**	❑ Resident is alive, in the current LTCF<br>❑ Resident is alive, temporary discharged (no hospital)<br>❑ Resident is deceased	❑ Resident is alive, at the hospital<br>❑ Resident is alive, permanently discharged (no hospital)<br>❑ Resident status is unknown

Surveillance of healthcare-associated infections and antimicrobial use in European long-term care facilities (HALT)

One-year incidence of infections in long-term care residents: a prospective study

Resident Questionnaire

Resident identifiers

ResidentID	

Temporary discharges

Date of temporary discharge	Date of re-admission in the current LTCF	Place where discharged	Reason for the hospitalisation
		❑ Home ❑hospital urgent ❑ hospital planned ❑ other LTCF ❑ Unknown	❑ Medical ❑ Surgical ❑ Diagnostic procedures

Annex 3. Infection questionnaire

Surveillance of healthcare-associated infections and antimicrobial use in European long-term care facilities (HALT)

One-year incidence of infections in long-term care residents: a prospective study

HAI Questionnaire

Resident identifiers

ResidentID		

HAI

Infection site		
If OTHER, please specify		
Start date		
End date		
Infection outcome	❑ Alive<br>❑ Death as part of the causal sequence<br>❑ Death, no contribution<br>❑ Unknown status or not verified	❑ Death, sole cause<br>❑ Death as contributory cause<br>❑ Unknown or not verified
Infection diagnosed in hospital	❑ Yes ❑ No ❑ Unknown	

Microorganisms

Name of the **first** mm.o isolated		S/I/R
Tested antimicrobials and resistance		
Name of the **second** mm.o isolated		S/I/R
Tested antimicrobials and resistance		
Name of the **third** mm.o isolated		S/I/R
Tested antimicrobials and resistance		

Comments & notes

Report any comment and/or notes – this is for internal use, do not report in the electronic data collection tools

Annex 4. Case definitions of infections

One-year incidence of infections in long-term care facility residents: a longitudinal study (H4LS)

CASE DEFINITIONS OF INFECTIONS

IMPORTANT REMARK:

All **active infections** present during the follow-up period should be reported. An infection is **active** when signs/symptoms of the infection are present **OR** signs/symptoms were present in the past and the resident is (still) receiving treatment for that infection

* Fever: 1) single > 37.8°C oral/tympanic membrane or 2) repeated > 37.2°C oral or > 37.5°C rectal or 3) > 1.1°Cover baseline from any site (oral, tympanic, axillary)

** Leucocytosis: 1) Neutrophilia > 14,000 leucocytes/mm3 or 2) left shift (>6% bands or ≥ 1500 bands/mm3)

§ Acute change in mental status from baseline: Acute onset + fluctuating course + inattention AND either disorganized thinking or altered level of consciousness

§§ Acute functional decline: New 3 point increase in total ADL score (Range 0-28) from baseline based on 7 ADL items (bed mobility, transfer, locomotion, dressing, toilet use, personal hygiene, eating) each scored from 0 (independent) - 4 (total dependence) OR increased dependency defined by scales other than ADL

URINARY TRACT INFECTIONS

❒ Resident without a urinary catheter

SIGNS/SYMPTOMS

*AT LEAST **ONE** OF THE FOLLOWING (1, 2 or 3) CRITERIA:*

- ❒ 1 Acute dysuria OR acute pain/swelling or tenderness of the testes, epididymis, or prostate
- ❒ 2 Fever* OR leukocytosis**

AND

One or more of the following:

- ☐ Acute costovertebral angle pain
- ☐ Suprapubic pain/tenderness
- ☐ Gross hematuria
- ☐ New or marked increase in frequency
- ☐ New or marked increase in urgency
- ☐ New or marked increase in incontinence
- ❒ 3 Two or more (in the absence of fever or leucocytosis):

URINE CULTURE

- ❒ Not Done, negative or test results unknown
- ❒ Urine culture done AND:
  - ☐ At least 10^5 cfu/ml of no more than 2 species of micro-organisms in a voided urine sample

  OR

  - ☐ At least 10^2 cfu/ml of any number of organisms in a

❒ Resident with a urinary catheter

SIGNS/SYMPTOMS

*AT LEAST **ONE** OF THE FOLLOWING (1, 2, 3 or 4) CRITERIA:*

- ❒ 1 Fever*, rigors, OR new onset hypotension with NO alternate site of infection
- ❒ 2 Acute change mental status **§** OR acute functional decline **§§** with NO alternate diagnosis AND leukocytosis**
- ❒ 3 New onset suprapubic or costovertebral angle pain or tenderness
- ❒ 4 Purulent discharge around catheter or acute pain, swelling or tenderness of testes, epididymis, or prostate

URINE CULTURE

- ❒ Not done, negative or test results unknown
- ❒ Urine culture done AND:
  - ☐ At least 10^5 cfu/ml of any organism(s) in a urinary catheter specimen

INFECTION CONFIRMATION

- ❒ Signs/symptoms AND urine culture positive: *INFECTION **CONFIRMED*** *(= **UTI-C**)*
- ❒ Signs/symptoms AND urine culture not done, negative or results unknown: *INFECTION **PROBABLE*** *(= **UTI-P**)*

RESPIRATORY TRACT INFECTIONS

COMMON COLD or PHARYNGITIS

*AT LEAST **TWO** OF THE FOLLOWING CRITERIA*:

- ❐ Runny nose or sneezing
- ❐ Stuffy nose (i.e. congestion)
- ❐ Sore throat or hoarseness or difficulty in swallowing
- ❐ Dry cough
- ❐ Swollen or tender glands in the neck (cervical lymphadenopathy)

INFECTION CONFIRMATION

❐ Infection criteria fully met: *INFECTION **CONFIRMED*** (= **COLD**)

***FLU** diagnosis can be made also outside the Flu season*

***BOTH** OF THE FOLLOWING CRITERIA MUST BE MET:*

❐ Fever *(for definition see top of page 1)*
AND

❐ At least **three** of the following:
- ☐ Chills
- ☐ New headache or eye pain
- ☐ Myalgias or body aches
- ☐ Malaise or loss of appetite
- ☐ Sore throat
- ☐ New or increased dry cough

INFECTION CONFIRMATION

❐ Infection criteria fully met: *INFECTION **CONFIRMED*** (= **FLU**)

LOWER RESPIRATORY TRACT INFECTIONS

❐ Resident **with** a POSITIVE chest x-ray for pneumonia or a new infiltrate

SIGNS/SYMPTOMS

***BOTH** OF THE FOLLOWING CRITERIA MUST BE MET:*

❐ At least **one** of respiratory signs or symptoms:
- ☐ New or increased cough
- ☐ New/increased sputum production
- ☐ 0_2 saturation < 94% or reduced >3% from baseline
- ☐ Abnormal lung examination (new or changed)
- ☐ Pleuritic chest pain
- ☐ Respiratory rate ≥ 25 breaths/min

AND

❐ One or more constitutional signs/symptoms (fever, leucocytosis, confusion, acute functional decline; *for definitions see top of page 1* **§§**)

❐ Resident **without** a POSITIVE chest x-ray for pneumonia or a new infiltrate OR chest x-ray not done

SIGNS/SYMPTOMS

***BOTH** OF THE FOLLOWING CRITERIA MUST BE MET:*

❐ At least **two** of respiratory signs or symptoms:
- ☐ New or increased cough
- ☐ New/increased sputum production
- ☐ O2 saturation < 94% or reduced >3% from baseline
- ☐ Abnormal lung examination (new or changed)
- ☐ Pleuritic chest pain
- ☐ Respiratory rate ≥ 25 breaths/min

AND

❐ One or more constitutional signs/symptoms (fever, leucocytosis, confusion, acute functional decline; *for definitions see top of page 1* **§§**)

Absence of other conditions such as chronic heart failure that could account for symptoms

INFECTION CONFIRMATION

❐ Signs/symptoms criteria met AND chest x-ray positive: *PNEUMONIA INFECTION **CONFIRMED** (= **PNEU**)*

INFECTION CONFIRMATION

❐ Infection criteria fully met: *OTHER LOWER RTI **CONFIRMED** (= **LRTI**)*

COVID-19

❒ The resident must have documentation in the medical record of any laboratory confirmation test for COVID-19 (viral RNA target or antigenic detection from an oropharyngeal or nasal swab or any other appropriate clinical specimen), or according to national definitions of infections in place at the moment of the follow-up. The infection should be reported even if there is no evidence of clinical signs or symptoms.

↓

❒ Asymptomatic (no signs and symptoms of any infection nor status alteration is reported)
❒ Mild/Moderate (any sign or symptom compatible with COVID-19*, without need for oxygen therapy and oxygen saturation level ≥ 92%)
❒ Severe (signs or symptoms compatible with COVID-19 with need for oxygen therapy for shortness of breath due to COVID-19 and/or oxygen saturation level <92%)

↓

INFECTION CONFIRMATION

❒ Infection criteria fully met: *INFECTION* ***CONFIRMED - Asymptomatic*** (= **COV-ASY**)
❒ Infection criteria fully met: *INFECTION* ***CONFIRMED – Mild/moderate*** (= **COV-MM**)
❒ Infection criteria fully met: *INFECTION* ***CONFIRMED - Severe*** (= **COV-SVR**)

↓

IMPORTANT FOR COVID-19 REPORTING

In case of a symptomatic infection, apply site specific case definition and, in case of confirmation, report as a separate infection indicating VIRCOV as microorganism.

*Signs and symptoms compatible with COVID-19:

Fever, cough, fatigue, shortness of breath, anorexia, myalgias, loss of smell (anosmia), loss of taste (ageusia). Other non-specific symptoms, such as sore throat, nasal congestion, headache, diarrhoea, nausea and vomiting, have also been reported.

Additional neurological manifestations reported include dizziness, agitation, weakness, seizures, or findings suggestive of stroke including trouble with speech or vision, sensory loss, or problems with balance in standing or walking.

Older people and immunosuppressed patients in particular may present with atypical symptoms such as fatigue, reduced alertness, reduced mobility, diarrhoea, loss of appetite, confusion, and absence of fever.

SURGICAL SITE INFECTIONS

Infection occurs within 30 days after the operation if no implant is left in place, or within three months if implant is in place

SUPERFICIAL INCISIONAL

__BOTH__ OF THE FOLLOWING CRITERIA MUST BE MET:

- ❒ Infection involves only skin and subcutaneous tissue of the incision

AND

- ❒ At least **one** of the following:
  - ☐ Purulent drainage with or without laboratory confirmation, from the superficial incision
  - ☐ Organisms isolated from an aseptically obtained culture of fluid or tissue from the superficial incision
  - ☐ At least one of the following signs or symptoms of infection:
    - ☐ Tenderness or pain
    - ☐ Localised swelling
    - ☐ Redness
    - ☐ Heat
  - ☐ Diagnosis of superficial incisional SSI made by a surgeon or attending physician

INFECTION CONFIRMATION

❒ Infection criteria fully met: *INFECTION **CONFIRMED*** (= **SSI-S**)

DEEP INCISIONAL

__BOTH__ OF THE FOLLOWING CRITERIA MUST BE MET:

- ❒ Infection appears to be related to the operation and infection involves deep soft tissue (e.g. fascia, muscle) of the incision

AND

- ❒ At least **one** of the following:
  - ☐ Purulent drainage from the deep incision but not from the organ/space component of the surgical site
  - ☐ A deep incision spontaneously dehisces or is deliberately opened by a surgeon when the patient has at least one of the following signs or symptoms: fever (> 38 °C), localised pain or tenderness, unless incision is culture-negative.
  - ☐ An abscess or other evidence of infection involving the deep incision is found on direct examination, during reoperation, or by histopathologic or radiologic examination.
  - ☐ Diagnosis of deep incisional SSI made by a surgeon or attending physician

INFECTION CONFIRMATION

❒ Infection criteria fully met: *INFECTION **CONFIRMED*** (= **SSI-D**)

ORGAN/SPACE

__BOTH__ OF THE FOLLOWING CRITERIA MUST BE MET:

- ❒ Infection appears to be related to the operation and infection involves any part of the anatomy (e.g. organs and spaces) other than the incision which was opened or manipulated during an operation

AND

- ❒ At least **one** of the following:
  - ☐ Purulent drainage from a drain that is placed through a stab wound into the organ/space
  - ☐ Organisms isolated from an aseptically obtained culture of fluid or tissue in the organ/space
  - ☐ An abscess or other evidence of infection involving the organ/space that is found on direct examination, during reoperation, or by histopathologic or radiologic examination
  - ☐ Diagnosis of organ/space SSI made by a surgeon or attending physician

INFECTION CONFIRMATION

❒ Infection criteria fully met: *INFECTION **CONFIRMED*** (= **SSI-O**)

NOTE:

If the infection matches one of the Surgical Site Infection (SSI) definitions, please give priority to the SSI. Do not apply another case definition for the same infection.

SKIN INFECTIONS

CELLULITIS/SOFT TISSUE/WOUND INFECTIONS

***ONE** OF THE FOLLOWING (*1 or 2*) CRITERIA MUST BE MET:*

- ❒ 1 Pus at a wound, skin, or soft tissue site
- ❒ 2 Four or more new or increasing signs/symptoms at affected site:
  - ❒ Heat
  - ❒ Tenderness or pain
  - ❒ Redness
  - ❒ Serous drainage
  - ❒ Swelling
  - ❒ One constitutional sign/symptom (fever, leucocytosis, confusion, acute functional decline; *for definitions see top of page 1)*

↓

INFECTION CONFIRMATION

Infection criteria fully met: *INFECTION **CONFIRMED***

(= **SKIN**)

SCABIES

***BOTH** OF THE FOLLOWING CRITERIA MUST BE MET:*

- ❒ Maculopapular and/or itching rash

AND

- ❒ At least **one** of the following:
  - ❒ Physician diagnosis
  - ❒ Laboratory confirmation (positive scraping or biopsy)
  - ❒ Epidemiological linkage to a case of scabies with lab confirmation

↓

INFECTION CONFIRMATION

❒ Infection criteria fully met: *INFECTION **CONFIRMED***
(= **SCAB**)

HERPES SIMPLEX OR ZOSTER INFECTION

***BOTH** OF THE FOLLOWING CRITERIA MUST BE MET:*

- ❒ A vesicular rash

AND

- ❒ Physician diagnosis or laboratory confirmation

↓

INFECTION CONFIRMATION

❒ Infection criteria fully met: *INFECTION **CONFIRMED***
(= **HERP**)

FUNGAL INFECTION

***BOTH** OF THE FOLLOWING CRITERIA MUST BE MET:*

- ❒ Characteristic rash or skin lesions

AND

- ❒ Physician diagnosis or lab confirmed fungal pathogen from scraping or biopsy

↓

INFECTION CONFIRMATION

❒ Infection criteria fully met: *INFECTION **CONFIRMED***
(= **FUNG**)

EYE, EAR, NOSE AND MOUTH INFECTIONS

CONJUNCTIVITIS

***ONE** OF THE FOLLOWING (1, 2 or 3) CRITERIA MUST BE MET:*

- ❒ 1 Pus appearing from one or both eyes, present for at least 24 hours
- ❒ 2 New or increased conjunctival erythema, with or without itching
- ❒ 3 New or increased conjunctival pain, present for at least 24 hours

Symptoms must not be due to allergy or trauma to the conjunctiva

↓

INFECTION CONFIRMATION

❒ Infection criteria fully met: *INFECTION **CONFIRMED*** (= **CONJ**)

EAR

***ONE** OF THE FOLLOWING (1 or 2) CRITERIA MUST BE MET:*

- ❒ 1 Diagnosis by a physician of any ear infection
- ❒ 2 New drainage from one or both ears
- ❒ (non-purulent drainage must be accompanied by additional symptoms, such as ear pain or redness)

↓

INFECTION CONFIRMATION

❒ Infection criteria fully met: *INFECTION **CONFIRMED*** (= **EAR**)

SINUSITIS

❒ Sinusitis diagnosed by physician

↓

INFECTION CONFIRMATION

❒ Infection criteria fully met: *INFECTION **CONFIRMED*** (= **SINU**)

ORAL CANDIDIASIS

***BOTH** OF THE FOLLOWING CRITERIA MUST BE MET:*

❒ Presence of raised white patches on inflamed mucosa OR plaques on oral mucosa

AND

❒ Diagnosed by a dentist or a physician

↓

INFECTION CONFIRMATION

❒ Infection criteria fully met: *INFECTION **CONFIRMED*** (= **ORAL**)

GASTROINTESTINAL INFECTIONS

GASTROENTERITIS

***ONE** OF FOLLOWING (1, 2 or 3) CRITERIA MUST BE MET:*

- ❒ 1 Diarrhoea, three or more liquid or watery stools above normal baseline for the resident in 24-hr period
- ❒ 2 Vomiting, two or more episodes in 24-hr period
- ❒ 3 **Both** of the following:
  - ❒ Positive stool specimen for bacterial or viral pathogen

  AND
  - ❒ At least one of the following: nausea, vomiting, abdominal pain or tenderness, diarrhoea

INFECTION CONFIRMATION

❒ Infection criteria fully met: *INFECTION **CONFIRMED*** (= **GE**)

CLOSTRIDIOIDES (CLOSTRIDIUM) DIFFICILE INFECTION

***ONE** OF FOLLOWING (1, 2 or 3) CRITERIA MUST BE MET:*

- ❒ 1 Diarrhoeal stools or toxic megacolon **AND**
- ❒ a positive laboratory assay for *C. difficile* toxin A and/or B in stools or a toxin-producing *C. difficile* organism detected in stool via culture or other means e.g. a positive PCR result
- ❒ 2 Pseudomembranous colitis revealed by lower gastro-intestinal endoscopy
- ❒ 3 Colonic histopathology characteristic of *C. difficile* infection (with or without diarrhoea) on a specimen obtained during endoscopy or colectomy

INFECTION CONFIRMATION

❒ Infection criteria fully met: *INFECTION **CONFIRMED*** (= **CDI**)

BLOODSTREAM INFECTIONS

***ONE** OF THE FOLLOWING (1 or 2) CRITERIA MUST BE MET:*

- ❒ Two or more blood cultures positive for the same organism
- ❒ A single blood culture documented with an organism thought not to be a contaminant

AND

At least **one** of the following:

- ☐ Fever *(for definition see top of page 1)*
- ☐ New hypothermia (<34.5° C, or does not register on the thermometer being used)
- ☐ A drop in systolic blood pressure of >30 mm Hg from baseline
- ☐ Worsening mental or functional status

INFECTION CONFIRMATION

❒ Infection criteria fully met: *INFECTION **CONFIRMED*** (= **BSI**)

UNEXPLAINED FEVER

❒ The resident must have documentation in the medical record of fever *(for definition see top of page 1)* on two or more occasions at least 12 hours apart in any 3-day period, with no known infectious or non-infectious cause

INFECTION CONFIRMATION

❒ Infection criteria fully met: *INFECTION **CONFIRMED*** (= **FUO**)

OTHER INFECTION(S)

Please specify (= **OTHER**)

Annex 5. Code list with microorganisms

INSTRUCTIONS

Report microbiological results that are available during the survey.

STEP ONE

Specify up to three isolated microorganisms, using the microorganism code list (see below).

If no microbiological result is available, one of the following options must be selected:

_NOEXA	EXAMINATION NOT DONE	No diagnostic sample taken; no microbiological examination done
_NA	RESULTS NOT AVAILABLE	The results of the microbiological examination are not available or cannot be found
_NONID	MICROORGANISM NOT IDENTIFIED	Evidence exists that a microbiological examination has been done, but the micro-organism cannot be correctly classified
_STERI	STERILE EXAMINATION	A microbiological examination has been done, but the result was negative (e.g. negative culture)

STEP TWO

For each reported microorganism, indicate the susceptibility using the table below.

Microorganism	Tested antibiotic[2]	Antimicrobial resistance			
Staphylococcus aureus (STAAUR)	Oxacillin (OXA)	Susceptible (S)	–	Resistant (R)	Unknown (U)
	Glycopeptides (GLY)	Susceptible (S)	Intermediate (I)	Resistant (R)	Unknown (U)
Enterococcus* species** (ENC)	Glycopeptides (GLY)	Susceptible (S)	Intermediate (I)	Resistant (R)	Unknown (U)
**Enterobacteriaceae[1]**, including: *Escherichia coli (ESCCOL)* *Klebsiella* species (KLE***) *Enterobacter* species (ENB***) *Proteus* species (PRT***) *Citrobacter* species (CIT***) *Serratia* species (SER***) *Morganella* species (MOGSPP)	Third-generation cephalosporins (C3G)	Susceptible (S)	Intermediate (I)	Resistant (R)	Unknown (U)
	Carbapenems (CAR)	Susceptible (S)	Intermediate (I)	Resistant (R)	Unknown (U)
Pseudomonas aeruginosa (PSEAER)	Carbapenems (CAR)	Susceptible (S)	Intermediate (I)	Resistant (R)	Unknown (U)
Acinetobacter baumannii (ACIBAU)	Carbapenems (CAR)	Susceptible (S)	Intermediate (I)	Resistant (R)	Unknown (U)

[1] Antimicrobial resistance markers are not collected for other Enterobacteriaceae (e.g. Hafnia spp., Salmonella spp., Shigella spp., Yersinia spp.) [2]OXA: susceptibility to oxacillin, or other marker of MRSA, such as cefoxitin, cloxacillin, dicloxacillin, flucloxacillin, meticillin; GLY: susceptibility to glycopeptides: vancomycin or teicoplanin; C3G: susceptibility to third-generation cephalosporins: cefotaxime, ceftriaxone, ceftazidime; CAR: susceptibility to carbapenems: imipenem, meropenem, doripenem.

CODE	NAME OF THE MICROORGANISM
	**- A -**
ACHSPP	ACHROMOBACTER SPECIES
ACIBAU	ACINETOBACTER BAUMANNII
ACICAL	ACINETOBACTER CALCOACETICUS
ACIHAE	ACINETOBACTER HAEMOLYTICUS
ACILWO	ACINETOBACTER LWOFFII
ACINSP	ACINETOBACTER SPECIES, *not specified*
ACIOTH	ACINETOBACTER SPECIES, *other*
ACTSPP	ACTINOMYCES SPECIES
AEMSPP	AEROMONAS SPECIES
AGRSPP	AGROBACTERIUM SPECIES
ALCSPP	ALCALIGENES SPECIES

CODE	NAME OF THE MICROORGANISM
ANANSP	ANAEROBES, *not specified*
ANAOTH	ANAEROBES, *other*
ASPFUM	ASPERGILLUS FUMIGATUS
ASPNIG	ASPERGILLUS NIGER
ASPNSP	ASPERGILLUS SPECIES, *not specified*
ASPOTH	ASPERGILLUS SPECIES, *other*
**- B -**	
GNBNSP	BACILLI, GRAM NEGATIVE, *not specified*
GNBOTH	BACILLI, GRAM NEGATIVE, NON ENTEROBACTERIACEAE, *other*
GPBNSP	BACILLI, GRAM POSITIVE, *not specified*
GPBOTH	BACILLI, GRAM POSITIVE, *other*
BACSPP	BACILLUS SPECIES
BCTOTH	BACTERIA, *other*
BATFRA	BACTEROIDES FRAGILIS
BATOTH	BACTEROIDES, *other*
BURCEP	BURKHOLDERIA CEPACIA
**- C -**	
CAMSPP	CAMPYLOBACTER SPECIES
CANALB	CANDIDA ALBICANS
CANGLA	CANDIDA GLABRATA
CANKRU	CANDIDA KRUSEI
CANPAR	CANDIDA PARAPSILOSIS
CANNSP	CANDIDA SPECIES, *not specified*
CANOTH	CANDIDA SPECIES, *other*
CANTRO	CANDIDA TROPICALIS
CHLSPP	CHLAMYDIA SPECIES
CITFRE	CITROBACTER FREUNDII
CITDIV	CITROBACTER KOSERI (EX. DIVERSUS)
CITNSP	CITROBACTER SPECIES, *not specified*
CITOTH	CITROBACTER SPECIES, *other*
CLODIF	CLOSTRIDIUM DIFFICILE
CLOOTH	CLOSTRIDIUM, *other*
GNCNSP	COCCI, GRAM NEGATIVE, *not specified*
GNCOTH	COCCI, GRAM NEGATIVE, *other*
GPCNSP	COCCI, GRAM POSITIVE, *not specified*
GPCOTH	COCCI, GRAM POSITIVE, *other*
CORSPP	CORYNEBACTERIUM SPECIES
**- E -**	
ENBAER	ENTEROBACTER AEROGENES
ENBAGG	ENTEROBACTER AGGLOMERANS
ENBCLO	ENTEROBACTER CLOACAE
ENBGER	ENTEROBACTER GERGOVIAE
ENBSAK	ENTEROBACTER SAKAZAKII
ENBNSP	ENTEROBACTER SPECIES, *not specified*
ENBOTH	ENTEROBACTER SPECIES, *other*
ETBNSP	ENTEROBACTERIACEAE, *not specified*
ETBOTH	ENTEROBACTERIACEAE, *other*
ENCFAE	ENTEROCOCCUS FAECALIS
ENCFAI	ENTEROCOCCUS FAECIUM
ENCNSP	ENTEROCOCCUS SPECIES, *not specified*

CODE	NAME OF THE MICROORGANISM
ENCOTH	ENTEROCOCCUS SPECIES, *other*
ESCCOL	ESCHERICHIA COLI
- F -	
FILOTH	FILAMENTS, *other*
FLASPP	FLAVOBACTERIUM SPECIES
FUNOTH	FUNGI, *other*
- G -	
GARSPP	GARDNERELLA SPECIES
- H -	
HAEINF	HAEMOPHILUS INFLUENZAE
HAEPAI	HAEMOPHILUS PARAINFLUENZAE
HAENSP	HAEMOPHILUS SPECIES, *not specified*
HAEOTH	HAEMOPHILUS SPECIES, *other*
HAFSPP	HAFNIA SPECIES
HELPYL	HELICOBACTER PYLORI
- K -	
KLEOXY	KLEBSIELLA OXYTOCA
KLEPNE	KLEBSIELLA PNEUMONIAE
KLENSP	KLEBSIELLA SPECIES, *not specified*
KLEOTH	KLEBSIELLA SPECIES, *other*
- L -	
LACSPP	LACTOBACILLUS SPECIES
LEGSPP	LEGIONELLA SPECIES
LISMON	LISTERIA MONOCYTOGENES
- M -	
MORCAT	MORAXELLA CATHARRALIS
MORNSP	MORAXELLA SPECIES, *not specified*
MOROTH	MORAXELLA SPECIES, *other*
MOGSPP	MORGANELLA SPECIES
MYCATY	MYCOBACTERIUM, atypical
MYCTUB	MYCOBACTERIUM TUBERCULOSIS COMPLEX
MYPSPP	MYCOPLASMA SPECIES
- N -	
NEIMEN	NEISSERIA MENINGITIDIS
NEINSP	NEISSERIA SPECIES, *not specified*
NEIOTH	NEISSERIA SPECIES, *other*
NOCSPP	NOCARDIA SPECIES
- P -	
PAROTH	PARASITES, *other*
PASSPP	PASTEURELLA SPECIES
PRESPP	PREVOTELLA SPECIES
PROSPP	PROPIONIBACTERIUM SPECIES
PRTMIR	PROTEUS MIRABILIS
PRTNSP	PROTEUS SPECIES, *not specified*
PRTOTH	PROTEUS SPECIES, *other*
PRTVUL	PROTEUS VULGARIS
PRVSPP	PROVIDENCIA SPECIES
PSENSP	PSEUDOMONADACEAE FAMILY, *not specified*
PSEOTH	PSEUDOMONADACEAE FAMILY, *other*
PSEAER	PSEUDOMONAS AERUGINOSA

CODE	NAME OF THE MICROORGANISM
	**- S -**
SALENT	SALMONELLA ENTERITIDIS
SALNSP	SALMONELLA SPECIES, *not specified*
SALOTH	SALMONELLA SPECIES, *other*
SALTYM	SALMONELLA TYPHIMURIUM
SALTYP	SALMONELLA TYPHI or PARATYPHI
SERLIQ	SERRATIA LIQUEFACIENS
SERMAR	SERRATIA MARCESCENS
SERNSP	SERRATIA SPECIES, *not specified*
SEROTH	SERRATIA SPECIES, *other*
SHISPP	SHIGELLA SPECIES
STAAUR	STAPHYLOCOCCUS AUREUS
STAEPI	STAPHYLOCOCCUS EPIDERMIDIS
STAHAE	STAPHYLOCOCCUS HAEMOLYTICUS
STACNS	STAPHYLOCOCCI, COAGULASE-NEGATIVE, *not specified*
STAOTH	STAPHYLOCOCCI, COAGULASE-NEGATIVE (CNS), *other*
STANSP	STAPHYLOCOCCUS SPECIES, *not specified*
STEMAL	STENOTROPHOMONAS MALTOPHILIA
STRHCG	STREPTOCOCCAE, HAEMOLYTIC (C, G), *other*
STRAGA	STREPTOCOCCUS AGALACTIAE (B)
STRPNE	STREPTOCOCCUS PNEUMONIAE
STRPYO	STREPTOCOCCUS PYOGENES (A)
STRNSP	STREPTOCOCCUS SPECIES, *not specified*
STROTH	STREPTOCOCCUS SPECIES, *other*
	**- V -**
VIRADV	ADENOVIRUS
VIRCMV	CYTOMEGALOVIRUS (CMV)
VIRCOV	SARS-CORONAVIRUS-2
VIRENT	ENTEROVIRUS (POLIO, COXSACKIE, ECHO)
VIRHAV	HEPATITIS A VIRUS
VIRHBV	HEPATITIS B VIRUS
VIRHCV	HEPATITIS C VIRUS
VIRHIV	HUMAN IMMUNODEFICIENCY VIRUS (HIV)
VIRHSV	HERPES SIMPLEX VIRUS
VIRINA	INFLUENZA A VIRUS
VIRINB	INFLUENZA B VIRUS
VIRINC	INFLUENZA C VIRUS
VIRNOR	NOROVIRUS
VIRPIV	PARAINFLUENZAVIRUS
VIRRHI	RHINOVIRUS
VIRROT	ROTAVIRUS
VIRRSV	RESPIRATORY SYNCYTIAL VIRUS (RSV)
VIRVZV	VARICELLA-ZOSTER VIRUS
VIRNSP	VIRUS, *not specified*
VIROTH	VIRUS, *other*
	**- Y -**
YEAOTH	YEASTS, *other*
YERSPP	YERSINIA SPECIES
